# The Activation-Induced Assembly of an RNA/Protein Interactome Centered on the Splicing Factor U2AF2 Regulates Gene Expression in Human CD4 T Cells

**DOI:** 10.1371/journal.pone.0144409

**Published:** 2015-12-07

**Authors:** Thomas C. Whisenant, Eigen R. Peralta, Lauren D. Aarreberg, Nina J. Gao, Steven R. Head, Phillip Ordoukhanian, Jamie R. Williamson, Daniel R. Salomon

**Affiliations:** 1 Department of Molecular and Experimental Medicine, The Scripps Research Institute, La Jolla, California, United States of America; 2 Department of Integrative Structural and Computational Biology, The Scripps Research Institute, La Jolla, California, United States of America; 3 NGS and Microarray Core Facility, The Scripps Research Institute, La Jolla, California, United States of America; University of Crete, GREECE

## Abstract

Activation of CD4 T cells is a reaction to challenges such as microbial pathogens, cancer and toxins that defines adaptive immune responses. The roles of T cell receptor crosslinking, intracellular signaling, and transcription factor activation are well described, but the importance of post-transcriptional regulation by RNA-binding proteins (RBPs) has not been considered in depth. We describe a new model expanding and activating primary human CD4 T cells and applied this to characterizing activation-induced assembly of splicing factors centered on U2AF2. We immunoprecipitated U2AF2 to identify what mRNA transcripts were bound as a function of activation by TCR crosslinking and costimulation. In parallel, mass spectrometry revealed the proteins incorporated into the U2AF2-centered RNA/protein interactome. Molecules that retained interaction with the U2AF2 complex after RNAse treatment were designated as “central” interactome members (CIMs). Mass spectrometry also identified a second class of activation-induced proteins, “peripheral” interactome members (PIMs), that bound to the same transcripts but were not in physical association with U2AF2 or its partners. siRNA knockdown of two CIMs and two PIMs caused changes in activation marker expression, cytokine secretion, and gene expression that were unique to each protein and mapped to pathways associated with key aspects of T cell activation. While knocking down the PIM, SYNCRIP, impacts a limited but immunologically important set of U2AF2-bound transcripts, knockdown of U2AF1 significantly impairs assembly of the majority of protein and mRNA components in the activation-induced interactome. These results demonstrated that CIMs and PIMs, either directly or indirectly through RNA, assembled into activation-induced U2AF2 complexes and play roles in post-transcriptional regulation of genes related to cytokine secretion. These data suggest an additional layer of regulation mediated by the activation-induced assembly of RNA splicing interactomes that is important for understanding T cell activation.

## Introduction

T cell activation is a central mechanism of the mammalian adaptive immune response to pathogenic stimuli. Beginning with detection of antigen by the T cell receptor (TCR), the ensuing activation response is mediated by initiation of signaling cascades, transcription factor activation and translocation to the nucleus, immune-related gene expression changes, receptor display at the cell surface and cytokine secretion [1–3]. The gene expression changes that occur as a result of transcription factor activation and binding are recognized as critical to the downstream immune response and lineage specification of the T helper effector subsets such as Th1, Th2, Th17, and Treg [4]. Albeit less well characterized, post-transcriptional regulation, a term collectively representing pre-mRNA splicing, alternative splicing, polyadenylation, nonsense-mediated decay, nuclear export, mRNA transcript stability, ribosomal loading, and mRNA degradation, is also likely to be important in the context of immunity and T cell activation [5].

Global mRNA detection technologies have been used to measure expression and alternative splicing in various models of the immune response. Utilizing exon level microarrays, splicing has been measured in activation of human primary T cells [6] and primary T and B cells [7]. Alternative splicing has been extensively characterized using RNAseq in both a T cell line (JSL1) and primary CD4 peripheral T cells [8–10]. Moreover, in a survey of 60 immune-related genes that undergo alternative splicing during activation of CD4 T cells, 7 different RNA binding proteins were linked to specific alternative splicing events [9]. A recent paper identified the role of the splicing factor, CELF2, in regulating alternative splicing after PMA activation of the JSL1 leukemic CD4 T cell line and employed several similar approaches to our present work including sequencing after CELF2 knockdown to identify splicing changes [11]. A granular gene-level bottom-up approach is crucial to understanding the detailed mechanism of regulation of splicing of individual genes in T cell activation. Alternatively, a top-down approach involves examining the global changes in mRNA splicing that are regulated by activation-induced RNA-binding protein complexes and is going to produce different kinds of data from the current rigorous bottom-up approaches focused on the RNA biology of specific protein-protein and RNA-protein interactions. As such, we will explore the value contributed by top-down studies in the context of the role in CD4 T cell activation-induced gene expression that includes changes in both canonical and alternative splicing.

T-cell activation involves upregulating a very large suite of gene expression patterns, which in turn necessitates a general increase in splicing. Therefore, it is important to look at post-transcriptional regulation in addition to the upstream processes of signaling and transcription factor activation that initiate gene expression changes. Recently, post-transcriptional regulation of the immune response has been studied in the context microRNA-mediated modulation of molecular networks responsible for T-cell activation and differentiation [12, 13]. The premise of the present work is that post-transcriptional regulation by specific RNA binding proteins serves as another layer of regulation that is required due to the complexity and importance of the immune response. We began with investigating the essential splicing factor, U2AF2, during the activation of primary human CD4 T cells.

The U2AF heterodimer is composed of 35 kDa U2AF1 and 65 kDa U2AF2 subunits [14], and the interaction occurs via the U2AF Ligand Motif (ULM) on U2AF2 and the U2AF Homology Motif (UHM) on U2AF1 [15]. Canonically, the U2AF heterodimer recruits the components of the spliceosome to the 3’ splice site (3’ SS), where U2AF2 binds to the polypyrimidine tract, and U2AF1 binds to the dinucleotide AG motif at the 3’ end of the intron [16, 17]. A recent publication that applied the high throughput techniques RNAseq and CLIPseq, demonstrated the importance of the U2AF heterodimer in defining the 3’SS via binding to the polypyrimidine tract and subsequently regulating alternative splicing [18]. As a result, the U2AF heterodimer is involved in most splicing events of canonical and alternative exons. In T cells, very little is known about its protein-protein interactome, the function associated with these interactions, or the process that leads to their assembly with the U2AF heterodimer on RNA transcripts. Mutations in the 3’ SS can lead to exon skipping and has been associated with Autoimmune Lymphoproliferative Syndrome (ALPS) and other genetic diseases [19, 20]. Additionally, altered splicing caused by somatic mutations in U2AF1 has been linked to various cancers [21]. Impairing exon definition at the 3’SS by mutating either the splice site or the RNA-binding protein has important biological and health ramifications.

Another level of regulation involves recruitment of U2AF2 to the 3’ splice site via interactions with a class of RNA-binding proteins called splicing factors that act as enhancers or suppressors for splicing of exons proximal to their binding site [22–24]. It is through either binding to the U2AF heterodimer or blocking access to the 3’ splice site that many alternative splice mRNA variants are generated [25–28]. Functionally, U2AF2 has also been shown to bind spliced mRNAs [29] and play a role in 3’ end processing [30].

Here we use the central role of U2AF2 in 3’SS definition as an avenue to understand the regulation of changes in gene expression during CD4 T cell activation. First, we present a primary CD4 T cell culture protocol that combines the expandability of a T cell line with the ability to model primary T cell activation by measuring cytokine secretion and activation markers. RNA binding protein immunoprecipitation and RNA sequencing (RIPseq) was used to identify the activation-induced mRNA interactome bound by U2AF2. We then mapped these transcripts to specific immune pathways. Next, we used mass spectrometry proteomics to identify the activation-induced assembly of a unique protein interactome centered on U2AF2. The biological significance of the interactome was demonstrated by knocking down 4 selected members. The impact of these knockdowns on cytokine production and gene expression, including canonical and alternative splicing, revealed some of the crucial roles for activation-induced assembly of the U2AF2 interactome in important and different immune gene pathways. Our work provides a list of candidates and a foundation for future study into: 1) understanding how T cell activation drives the composition of the different splicing complexes and, 2) discovering the rules involved in T cell activation that initiate and guide the function of these complexes to regulate canonical and alternative splicing of specific genes.

## Methods

### Ethics, consent and permissions

All the studies in this manuscript were covered by Human Subjects Research Protocols approved by the Institutional Review Board of The Scripps Research Institute. Informed written consent was obtained from all study subjects.

### CD4+ T cell culture

250ml of peripheral blood from a normal human female was separated through a Ficoll-histopaque (Sigma) gradient to obtain peripheral blood mononuclear cells (PBMCs). CD4+ T cells were isolated from the PBMCs using Human CD4 Naïve Separation Kit (Stem Cell Technologies). The purified cells were transferred to RPMI 1640 culture medium (+ 10% FBS, 100U/ml Penicilin, Mediatech) at 37°C and 5% CO_2_, activated with Dynabead Human T-Activator CD3/CD28 (Invitrogen) for 48h, and cultured in the presence of 30 U/ml of IL2. The cells were expanded for 7 days at a density between 0.5x10^6^ to 2x10^6^ cells/ml. Aliquots of 40x10^6^ cells were collected in freezing media (90% FBS/10% DMSO) for storage at -80°C. Frozen cell aliquots were thawed for further expansion by culturing in RPMI supplemented with IL2 for an additional 7 days, then split in two groups for a 24h re-stimulation with T-activator beads or no stimulation (“resting”).

### Cell line and culture conditions

Jurkat cells, clone E6-1 (ATCC TIB 152 human acute T-cell leukemia) [31] were cultured in RPMI 1640 culture medium (+10% FBS, 100 U/mL penicillin) at 37°C and 5% CO_2_. Subculturing was performed every 2–3 days and fresh media was replaced 24 hours prior to each experiment. Resting and activated cells were, respectively, incubated without or with Dynabead Human T-Activator CD3/CD28 (Invitrogen) beads for 48h and collected for analysis.

### Flow Cytometry

Cell purity was assessed by flow cytometry staining with antibodies specific for CD4 (SK3, eBioscience), CD45RA (HI100, eBioscience), CD45RO (UCHL1, eBioscience). Assessment of activation markers was conducted with antibodies to CD25 (BC96, eBioscience), CD69 (FN50, Biolegend), CD62L (DREG-56, Biolegend), CD40L (24–31, eBioscience), CD71 (OKT-9, eBioscience). Data was acquired on an LSRII (BD Biosciences) and analyzed in FlowJo (Treestar).

### Cytokine ELISAs

Supernatants from cultured cells were collected and stored at -80°C. Cytokine expression from thawed supernatants was determined using the Q-Plex Human Cytokine Screen IR (16-plex, Quansys) following manufacturer’s instructions. Plates were read on a LI-COR Odyssey imaging system at multiple scanning intensities. Data was analyzed using Q-View Software (Quansys). Cytokine expression was background corrected by subtracting any signal from media only samples and data points below zero were removed. Technical replicates were averaged to yield a single value for analysis and ELISAs were performed in triplicate where each data point reflects a biological replicate. To compare means in siRNA knockdown experiments, we first log-transformed the data to meet the requirements of the statistical test. We then performed unpaired two-tailed Student’s t-tests where significance was defined as p < 0.05.

### MTT Assay

Proliferation of cultured primary CD4 T cells was measured using the MTT Cell Proliferation Assay (ATCC) following manufacturer’s instructions. Briefly, 80,000 cells were plated in 90 μl of media in flat bottom plates in the presence or absence of T-activator beads. After 24h, 10 μl of MTT reagent was added to each well. Plates were incubated at 37°C in a 5% CO_2_ incubator for 4 h. 100 μl of detergent reagent was added to each well and plates were incubated in the dark at room temperature overnight. Absorbance was measured at 570 and 650 nm. During analysis, the 650 nm absorbance and media alone controls were subtracted from each sample.

### ImmuneMap

ImmuneMap is composed of 54 immune-related pathways, each of which is made up of all genes known to be associated with the pathway directly at the protein level or indirectly via transcriptional regulation. A custom perl script was used to identify pathways (and their genes) and statistically determine each pathway’s enrichment for the gene list by hypergeometric testing.

### RNA Isolation, RNAseq, and Quantitative PCR

Cell aliquots were resuspended in Buffer RLT from the Rneasy purification kit (Qiagen) and total RNA was purified according to the manufacturer’s protocol. DNA Digestion was performed on the columns with the RNase-Free DNase Set (Qiagen). RNA concentration was determined using the RNA broad range assay on the Qubit Fluorometer (Invitrogen) and Quality control analysis was performed using Eukaryote Total RNA Nano chip on the 2100 Bioanalyzer (Agilent). For RNAseq, libraries were made using the Illumina TruSeq RNA Sample Preparation Kit preceded by a PolyA selection step. RNA sequencing was conducted on the HiSeq 2000 instrument (Illumina), generating 100-base paired-end reads. For quantitative PCR, 1 ug RNA was used as input in a cDNA synthesis reaction using SuperScript III (Thermo Fisher Scientific, Inc.) according to the manufacturer’s protocol.

### Sequence Mapping and Analysis

Sequencing and post-processing was performed by the TSRI Next Gen Sequencing Core Facility. The Genome Analyser Pipeline Software (Casava v1.8.2) was used to perform the preliminary data analysis of image processing and base calling. Output FASTQ files were filtered by quality score (Q > 25). Reads were mapped to hg19, downloaded from the UCSC Genome Browser (http://hgdownload.cse.ucsc.edu/), using TopHat version 2.0.9 [32] with a specified GTF file downloaded from the TopHat website (http://ccb.jhu.edu/software/tophat/igenomes.shtml). Aligned reads were filtered for mapping quality (q > 20) using SAMtools (http://samtools.sourceforge.net/) and optical duplicates using the markDuplicates tool from within the Picard suite of command-line utilities (http://picard.sourceforge.net/). The resulting BAM files were input to the R environment (http://cran.us.r-project.org/) where the reads were assigned to genes using the summarizeOverlaps function within the GenomicRanges package (http://www.bioconductor.org/packages/release/bioc/html/GenomicRanges.html). Gene level read counts were converted to FPKM (Fragment Per Kilobase of exon model per Million mapped reads, [33]) and genes with an FPKM > -2 for more than half of the samples in at least one group were retained for further analysis [34]. The filtered gene level read counts for each sample were used as input into the edgeR package [35] to measure differential gene expression with the threshold for significance set at an FDR adjusted p-value below 0.05. Heatmaps of differentially expressed genes were generated using the heatmap.2 package within R after normalizing read counts across samples and genes. Alternative splicing analysis was performed using AltAnalyze (http://www.altanalyze.org/) [36] which uses BAM files as input for the analysis. Using the ASPIRE algorithm as the metric, we used the default threshold (ASPIRE > 0.2) to determine differential splicing between two groups. AltAnalyze also uses GO-Elite to perform enrichment analysis on numerous ontologies and annotation databases including GeneOntology, KEGG, Biomarkers, and microRNA Targets. An immune specific ontology developed in house called ImmuneMap, was used to identify significantly enriched immune related pathways and functions contained within the lists of differentially expressed and/or alternatively spliced genes. Significantly enriched immune pathways were identified as those whose adjusted p-values (threshold less than 0.05) were calculated using a hypergeometric distribution.

### U2AF2 RNA Binding Protein Immunoprecipitation (RIP)

Cell pellets (~50x10^6^ cells) were resuspended in 0.5ml RIPA buffer (Sigma) supplemented with RNaseOUT (Invitrogen), Complete EDTA-free protease inhibitor cocktail tablets (Roche, Inc.), and the phosphatase inhibitors Sodium Fluoride (Sigma) and Sodium Orthovanadate (Sigma). After sonication (3x15” @ 70% power, Misonix Sonicator 3000), insoluble material was pelleted by centrifugation (12,000xg for 15’ @ 4°C), supplemented with 0.5ml NET-2 buffer (50mM Tris-HCl, pH 7.0; 150mM NaCl; 1mM MgCl_2_; 0.05% Igepal-CA 630; 1mM DTT, 1.5mM EDTA, RNaseOUT, protease inhibitor, phosphatase inhibitor), and quantified by BCA Assay (Pierce). Lysates were pre-cleared for 1h at 4°C with mouse IgG-conjugated Protein G beads (Invitrogen), lysates were incubated with a mouse monoclonal U2AF2 antibody (U4758, Sigma) conjugated to Protein G beads (rotating overnight @ 4°C). The beads were then washed 5x with Net-2 buffer. Where specified, RNAse A digestion was performed on the beads (10 ug/ml for 30 minutes at RT). After the washes, RNA/Protein complexes were eluted by resuspending the beads in SDS elution buffer (2% SDS in TE pH 8.0), and incubating for 30 minutes at 55°C. The elution was repeated a second time, and RNA from the combined eluates was extracted by performing an initial separation with Chloroform and Trizol LS (Invitrogen). The aqueous phase was then transferred to a new tube, and 3 volumes of 100% ethanol was added. After vortexing, the samples were loaded onto Rneasy columns (Qiagen) and RNA was purified according to the manufacturer’s instructions.

### RIPseq

The eluate from U2AF2 RIP (Resting and Activated, n = 6/group) was used as input into a TRIZOL separation and the RNA fraction was isolated washed, precipitated, and controlled for quality. 50ng of RNA treated with the RNase Free DNase Set (Qiagen) was used as input into the Script-Seq v2 RNA-Seq Library Preparation kit (Epicentre). The protocol was performed according to the manual with the following exceptions: 1) No PolyA purification or rRNA depletion steps were performed; 2) DNA Clean and Concentrator-5—PCR/DNA clean columns (Zymo Research) were used to isolate the cDNA before the PCR step. Single end 1x100 sequencing and post-processing were performed by the TSRI Next Gen Sequencing Core Facility. Downstream bioinformatic analysis was performed as described previously for RNAseq and the results are shown in S4 Table. Comparisons to RNAseq were made using calculated fpkm values.

### Mass Spectrometry

The eluate from U2AF2 RIP was used as input into a TRIZOL LS separation and the protein fraction isolated, washed and pelleted. Protein pellets (~15ug) were resuspended in 40ul of 100mM Ammonium Bicarbonate + 5% Acetonitrile (ACN) and sequentially incubated with 4ul of 50mM DTT (10’ @ 65°C), 4ul of 100mM Iodoacetamide (30’ @ 30°C) and 4ul of 400ng of Trypsin (4hrs @ 37°C without light). The digested samples were run over C-18 spin columns (Thermo), vacuum dried, and resuspended in 10ul 5% ACN + 0.1% Formic Acid. Peptides were loaded onto cHiPLC Nanoflex system (Eksigent) with an analytical separation column packed with ChromXP C_18_ (3 μm, 120 Å) and eluted off a linear gradient of 5–35% Solvent B at a flow rate of 0.4ul/min over 60’ (Solvent A: 0.1% (v/v) formic acid in water; Solvent B: 0.1% (v/v) formic acid in acetonitrile). Analysis was performed on the TripleTOF 5600 mass spectrometer (AB Sciex) with an electrospray ionization source in a mode where a single 250-ms TOF-MS survey scan was collected, from which the top 20 ions were selected for automated MS/MS 50-ms scans. Ions selected for MS/MS were excluded for a period of 20 seconds. Replicate output WIFF files (no RNase: n = 6/group; RNase treated: n = 4/group) were used as input into the software ProteinPilot (AB Sciex), which generates a list of identified proteins along with their associated false positive probability based on a series of searches through a reverse database. We retain the proteins below a threshold FDR of 1% then remove non-human hits and human hits from a compiled list of internally identified and known contaminant proteins in mass spectrometry (i.e. Keratins) [37].

### SILAC

Previously frozen CD4 T cells were thawed in deficient media (FBS/antibiotic supplemented RPMI 1640 lacking Lysine and Arginine amino acids). The remaining viable cell population was split in two, culturing each in media containing Lysine (0.274mM) and Arginine (1.15mM): 1) unlabeled (^14^N) Lysine and Arginine; 2) labeled (^15^N) Lysine and Arginine. Cells were expanded for an additional seven days when one of the randomly chosen groups was collected and frozen down as “resting” cells and the other group was stimulated with T-activator beads for 48h then frozen down as “activated”. Lysates were prepared from the frozen cell pellets and mixed based on initial cell number. These mixed lysates were used as input for U2AF2 RIP and subsequent protein preparation for the mass spectrometry protocol (described above). Analysis of the output files from mass spectrometry was performed as previously described [38, 39]. Briefly, an experimental peak list was compared to a theoretical digest of the human proteome. Additionally, ^14^N and ^15^N peaks corresponding to the same peptide were required to elute close to each other and exhibit the same charge state. Peaks with multiple identities were excluded. Isotope distributions were extracted for each of the identified ^14^N−^15^N peak pairs. Extracted mini-spectra were fit to theoretical isotope distributions using in-house software which implements a least squares Fourier transform convolution (LS-FTC) method. Resulting least-squares fits were visually inspected and filtered to eliminate data with low signal-to-noise ratio or spectral overlaps that are hard to resolve. The fit parameters for the ^14^N and ^15^N amplitudes were used for relative quantification of peptide levels and converted to ratios of resting/activated. Ratios for all peptides within a protein were log-transformed to meet to the requirements of the statistical test then used to calculate each mean and standard deviation. We used one-sample, two-tailed student’s t-test with multiple testing correction (H_o_ = 1, adjusted p-value < 0.05) to identify proteins with significant changes in U2AF2 association during activation (S5 Table).

### SDS-Page and Western Blot

Western blot analysis was performed using samples of whole cell lysate or eluate from immunoprecipitation prepared in NuPage sample buffer supplemented with antioxidant reagent according to manufacturer’s specifications (Invitrogen). Samples were heated (10’ @ 70°C), then separated by electrophoresis in NuPage 4–20% polyacrylamide gels and transferred to nitrocellulose membanes using the NuPage XCell II Blow Module (Invitrogen). Blots were visualized using the Li-COR Odyssey CLX fluorescent scanner after blocking with fluorescent blocking buffer (Li-COR Biosciences), incubation with primary antibodies and fluorescently conjugated secondary antibodies (Li-COR Biosciences).

Primary antibodies: Mouse monoclonal–HNRNPC1/C2 (sc-32308, Santa Cruz), HNRNPU (3C6, Millipore), SRSF3 (7B4, Millipore), SRSF1 (AK96, Millipore); Rabbit polyclonal–FUSIP1 (A302-282A-1,Bethyl), ILF2 (A303-147A-1, Bethyl), IgG (2729, Cell Signaling), U2AF1 (A302-079A-1, Bethyl), SAM68/KHDRBS1 (sc-333, Santa Cruz), SFRS10 (AV40528, Sigma).

### Transfection and siRNA-mediated knockdowns

Cells were transfected by Amaxa Nucleofection (Lonza) following the manufacturer’s instructions. Briefly, 4x10^6^ cells were pelleted and resuspended in 100 ul of supplemented SE cell line solution. siRNA was added to a final concentration of 300 nM. The cell suspension was then transferred to cuvettes and nucleofected using program CL-120. Cells were cultured for 48h prior to activation with Dynabead Human T-Activator CD3/CD28 (Invitrogen). After and additional 24h in culture, the cells were collected and flash frozen in liquid nitrogen. Cell pellets were subsequently used for RIP.

### siRNA-mediated knockdowns

siRNA was obtained from Qiagen (Table 1):

**Table 1:** Sequences and product numbers for target specific siRNA primers.

Target	Product	Sequence
Hs_U2AF1_7	SI04159547	CTGCTAGAAAGTGTTGTAGTT
Hs_SYNCRIP_7	SI04300604	CGCGGTAGAGCCGGTTATTCA
Hs_ILF2_7	SI03069129	CAGGACATGGTCTGCTATACA
Hs_SRRM2_4	SI00733460	CGCCACCTAAACAGAAATCTA

### Microarrays and RIP-Chip

Input into the PLUS kit (Affymetrix) was 1ug of total RNA (DNAse treated and passing quality control standards) from siRNA knockdown cells and, for RIP-Chip, 125ng of purified, DNAse treated, RNA from U2AF2 RIP. Labeled, fragmented cRNA was hybridized on the Human Transcriptome Array 2.0 and scanned to generate CEL files. CEL files from triplicate experiments were input into the Affymetrix Expression Console software to generate normalized, log-transformed RMA values. A log2(RMA) threshold of 6 was used to filter low and non-expressed genes prior to differential expression analysis by the limma package within the R environment (adjusted p-value < 0.05) [40, 41]. For differential splicing analysis, CEL files (n = 3/group) were used as input into the AltAnalyze package (as described above). Enrichment analysis was performed with GO-Elite (within AltAnalyze) for lists of differentially expressed and alternatively spliced genes as well as using the ImmuneMap ontology. When comparing Control siRNA to RNA-Binding Protein specific siRNA samples for specific junction and exon probes on the HTA 2.0, the Transcript Analysis Console (TAC, Affymetrix, Inc.) was used to calculate Splicing Index (SI) values.

### Availability of Supporting Data

The Microarray and next generation sequencing datasets supporting the results of this article are available in NCBI’s Gene Expression Omnibus (GEO, http://www.ncbi.nlm.nih.gov/geo/) and assigned the superseries accession number GSE62923. The mass spectrometry proteomics dataset supporting the results of this article to identify the U2AF2 interactome is available at the ProteomeXchange Consortium (http://www.proteomexchange.org/) [42] via the PRIDE partner repository with the dataset identifier PXD001843. The SILAC mass spectrometry proteomics dataset supporting the results of this article is available at the ProteomeXchange Consortium via the PRIDE partner repository with the dataset identifier PXD001846.

## Results

### Culture of primary CD4 T cells for studies of activation-induced splicing

Isolated primary CD4 T cells from normal human donors are a model for T cell activation, but the availability of primary human cells limits their use in large-scale genomic studies. To address this issue, a human CD4 T cell culture derived from primary cells was developed that preserves the changes in gene expression and cytokine secretion characteristic of T cell activation that are not observed in the commonly used model, cultured Jurkat T cells. Briefly, human primary CD4 T cells are stimulated with α-CD3/α-CD28 beads for 48 hours at the time of isolation. This is followed by a 7 day expansion, where the cells are cultured in the presence of IL2, then frozen and stored as reusable aliquots. An additional post-thaw 7 day expansion of the cells establishes a functional “resting” state from which they can be re-stimulated with α-CD3/α-CD28 beads to measure changes associated with re-“activation”.

**Fig 1:**
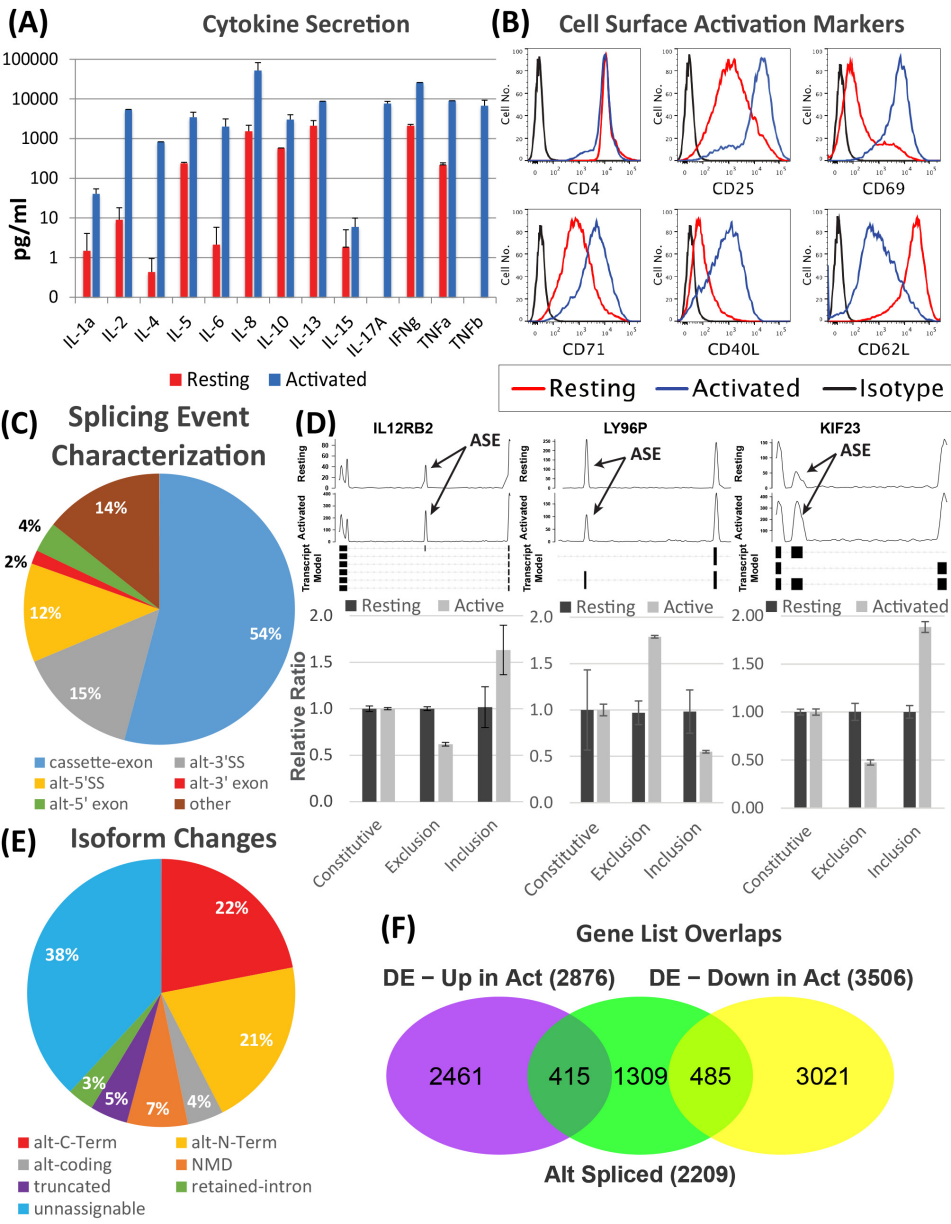
Protocol for primary T cell culture displays phenotypic markers and transcriptional changes indicative of activation. (A) ELISA results for secretion of 12 cytokines in resting and activated CD4 T cell cultures. Error bars represent mean ± S.E.M. (n = 3). (B) Histogram depicts expression of the T cell marker CD4 and various activation markers (CD25, CD69, CD71, CD62L, and CD40L) from FACS analysis of resting (red) and activated (blue) CD4 T cell cultures. **(**C) Breakdown of differential splicing events between resting and activated CD4 T cell culture based on RNASeq data using Alt-Analyze (ASPIRE > 0.2 & p-value < 0.1, S1 Table). **(**D) RT-qPCR validation of alternative splicing in RNAseq data for IL12RB2, LY96P, & KIF23. ASE–Alternatively Spliced Exon. Error bars represent mean ± S.D. (n = 3). (E) Breakdown of isoform changes by type between resting and activated T cells identified by Alt-Analyze in RNAseq data. (F) Three classes of gene expression are demonstrated after T cell activation: 1) DE, alone (5303); 2) DE+AS (1079); 3) AS, alone (1281). This data is also shown for DE—upregulated and DE—downregulated. DE–Differentially Expressed; AS–Alternatively Spliced.

Various T cell specific cytokines were measured using culture media collected from “Resting” and “Activated” cells. Significant post-activation increases were observed in 12 of 13 cytokines screened, representing most of the primary cytokines linked with immunity and inflammation (Fig 1A). In contrast, measurement of the same cytokine panel with media from stimulated Jurkat T cells showed little or no cytokine secretion beyond IL8 (S1A Fig). Using flow cytometry as an orthogonal measure of T cell activation, the expected changes in surface expression of CD25, CD40L, CD62L, CD69, and CD71 were demonstrated (Fig 1B). Similarly, activation of Jurkat cells resulted in increased expression of CD25, CD69 and the expected reduction in CD62L expression. However, upregulation of CD40L, and CD71 expression that characterizes activation of the CD4 T cell was not observed (S1B Fig).

To investigate activation-induced splicing and the gene expression networks underlying phenotypic changes observed with activation of our CD4+ T cell culture, the transcriptome was profiled by paired end RNAseq. After sequencing of two replicate samples in each of the two groups, the data was aligned to the human transcriptome and analyzed for differential expression and alternative splicing. The Bioconductor package edgeR [35] was used to generate lists of significantly differentially expressed genes (S1 Table). Of the differentially expressed genes, the distribution of up and downregulated are similar in magnitude (S2A Fig). Moreover, the 6382 genes that undergo activation-induced expression and splicing are nearly 50% of the 13,070 genes that pass our threshold level for detectable expression.

Next, activation-induced alternative splicing was profiled with Alt-Analyze using the ASPIRE algorithm [36]. This algorithm requires identification of two significant, independent splicing events that are correlated with a single isoform change making it a more stringent tool for identifying alternative splicing events. 2209 genes were identified with significant alternative splicing events using ASPIRE (S1 Table). Moreover, our RNAseq data confirmed the specific splicing events listed for 27 of 60 (45%) genes previously reported, and identified an additional set of alternative splice variants in 16 members of that set of genes. These results expand the current literature catalog of 60 validated alternative splicing events in CD4 T cells described previously [10].

Annotation of the differential splicing events by type is shown in Fig 1C. The predominant splicing event is the inclusion or exclusion of a cassette exon. 15 alternatively spliced genes were selected based on detection of cassette exon changes and RT-qPCR was performed, validating our results successfully in all selected genes (S2B Fig). Fig 1D shows the good correlation between qPCR and deep sequencing for the genes IL12RB2, LY96P, KIF23. Changes in the 5’ splice site (5’SS) and 3’ splice site (3’SS) are the next most common events, resulting in changes in the exon’s length. While many observed splicing events couldn’t be linked to a specific isoform change (unassignable), Fig 1E also shows the types of isoform switching that are detected. Of the assignable events, more than 43% represent increased or decreased splicing of regions at the N- or C-termini suggesting selection of alternative translational start and stop sites creating different protein isoforms. We used sashimi plots to bioinformatically validate these findings (S3 Fig and S4 Fig, respectively). Many alternatively spliced genes vacillate between two isoforms where a single splicing event correlates with a single isoform switch. Over 50% of genes have more than a single alternative splicing event. Unfortunately, the limitations of isoform annotations at this time make it difficult to assign all the splicing events detected by RNAseq to specific isoforms, exemplified by the 38% unassignable isoform changes. While these different classes of splicing events have been documented in other cell types, this work represents a significant addition to the current knowledge of alternative splicing in a primary CD4 T cell culture.

In our previous study of activation-induced alternative splicing in primary CD4 and CD8 T cells, we identified three classes of gene expression changes: differentially expressed, alternatively spliced and both differentially expressed and alternatively spliced [7]. In mapping the members of these three classes to functional pathways, it was shown that the class of genes that is both differentially expressed and alternatively spliced has an important role in various aspects of T cell activation including signal transduction, cytokine signaling, cell adhesion and cell cycle progression. In the present analysis, these same three classes were identified in the primary CD4 T cell cultures (Fig 1F) and two different mapping tools were used to confirm a similar enrichment for immune/inflammatory gene pathways in the class of differentially expressed/alternatively spliced genes. Using the GOSlim ontology, the four significantly enriched categories (Fisher Exact p <0.05, S2 Table) were cell division, immune system process, biological adhesion, and cell cycle. To search for enrichment of immune specific functions, we used an internally developed ontology tool called ImmuneMap and identified 6 significantly enriched pathways including T cell Receptor signaling, general chemokine, and costimulation pathways (S3 Table). These data support the conclusion that the phenotype of CD4 T cell activation is accompanied by profound changes in the transcriptome brought about by differential gene expression and alternative splicing, while confirming that our method of culturing primary cells is a relevant model for studying CD4 T cell activation.

### Changes in the activation-inducted transcriptome are reflected in transcript association with the splicing factor U2AF2

Activation-induced expression and changes in alternative splicing are mediated by splicing factors and components of the spliceosome, and to begin to characterize these changes, we sought to characterize the mRNAs and proteins associated with the major splicing factor U2AF2 in the context of T cell activation. The U2 Auxillary Factor (U2AF) heterodimer is comprised of a 35KDa protein, U2AF1 or U2AF35, and a larger 65KDa protein, U2AF2 or U2AF65. The U2AF heterodimer is responsible for binding the 3’SS and the adjacent 5’ region known as the polypyrimidine tract and then guiding the assembly of a series of protein complexes required for splicing [43]. Only recently has there been a genome-wide test of the generality of U2AF2s role in splicing using Cross-linking and ImmunoPrecipitation followed by deep sequencing (CLIPseq), where U2AF heterodimers were localized to 88% of known 3’ SS in a static system of the HeLa cell line [18]. Our objective was to determine if changes in expression profile and alternative splicing upon activation of CD4 T cells in culture, were reflected in U2AF2 bound transcripts, and to determine if those transcripts were overrepresented in immunologically relevant networks.

**Fig 2:**
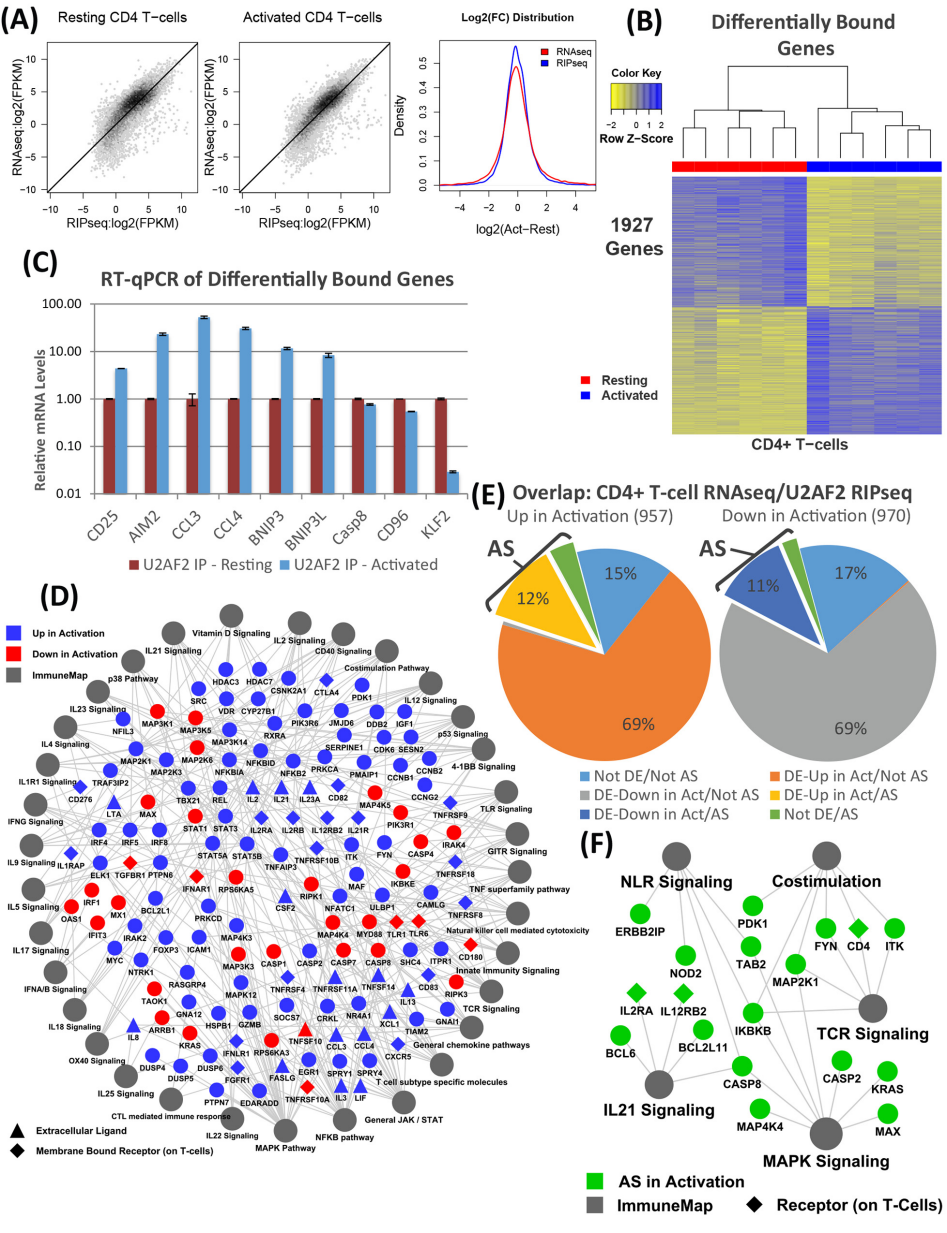
RIPseq identifies critical immune genes bound to and spliced by U2AF2 upon activation. (A) Hexbin plots comparing log_2_ (fpkm) values for U2AF2 RIPseq and RNAseq in resting (left) and activated (middle) CD4 T cell cultures. *Right*: Plot comparing the distribution of log_2_ (fold change) in RNAseq (red) and U2AF2 RIPseq (blue). (B) Heatmap of 1,927 significantly differentially bound genes in U2AF2 RIPseq (edgeR adjusted p-val < 0.05, n = 6/group). 957 increased and 970 decreased their binding to U2AF2 upon activation. (C) RT-qPCR of select differentially bound genes in U2AF2 RIPseq. Error bars represent mean ± S.D. (n = 3). (D) Cytoscape map of overlap of differentially expressed (upon activation) and differentially bound (to U2AF2) genes within significantly enriched ImmuneMap pathways. (E) Overlap of significantly differentially bound transcripts in U2AF2 RIPseq with differentially expressed & alternatively spliced genes in the RNAseq data. (F) Immune pathways significantly enriched (hypergeometric test adjusted p-value <0.05) for overlapping genes differentially bound during U2AF2 RIPseq and alternatively spliced (upon activation) in RNAseq data.

The first step was to determine the RNA targets of U2AF2 using RNA Binding Protein Immunoprecipitation (RIP) followed by next generation sequencing, RIPseq (n = 6, S5A Fig). CLIPseq is a protocol that was developed to identify peaks around the sequences bound by an RNA-binding protein and reveal the consensus RNA binding motif targeted by the RBP under study. In contrast, we have employed a RIPseq protocol with no crosslinking and a simple IP purification of the mRNA transcripts bound by our target RBPs. Our protocol allows us to map reads with increased coverage of each transcript, thus increasing confidence in identified transcripts associated with U2AF2 and suitable for mapping to functions and pathways. To eliminate non-specific binding of RNA and protein to the U2AF2 antibody, we pre-absorbed the cell lysate with affinity purified isotype control antibody (mouse IgG). In parallel experiments, RIPseq done with the mouse IgG control pulled down no detectable RNA. Comparing the U2AF2 RIPseq to the RNAseq done on whole cells, there is a slight reduction in the total number of genes detected, reflected by the compaction seen in the lower fpkm range (Fig 2A). However, this does not limit the ability to compare resting and activated samples, as seen in the normal distribution of log_2_(fpkm) values and the scatter plot comparing those values (S5B Fig).

A total of 10,716 genes from the U2AF2 RIPseq passed an experimentally determined expression threshold of log_2_(fpkm) >2 (S4 Table). Thus, the transcripts significantly “bound” by U2AF2 complexes accounts for 82% of the transcripts identified as significantly expressed by RNAseq (13,070 genes). Next, the changes in U2AF2-bound transcripts with activation were compared. A heatmap of the 1,927 significantly differentially “bound” transcripts is shown in Fig 2B, where 957 increase and 970 decrease their binding activation-dependent binding to U2AF2. The heatmap profile of up and down regulated transcripts is very similar to that obtained with RNAseq (S2A Fig). For validation, we chose 10 genes that were either at the top of the list in terms of significance by FDR-adjusted p-value and/or significant genes that were also immunologically interesting. RT-qPCR validated these genes with varying expression patterns (Fig 2C). The full network of genes that were both differentially bound and differentially expressed with activation and found in significantly enriched immune pathways is shown in Fig 2D, where nearly two-thirds increase upon activation. These 100 genes are connected to 33 ImmuneMap pathways with the most densely connected being MAPK, NFKB, and JAK/STAT signaling, T cell subset-specific and chemokine pathways.

Next, we further compared the activation-induced differentially U2AF2-bound genes with the differential expression and alternative splicing data obtained with RNAseq (Fig 2E). For the genes that either decrease or increase their binding to U2AF2 upon activation in the RIPseq data, ~80% are differentially expressed in the same direction in the RNAseq data; moreover, 11–12% of these genes are also alternatively spliced in the RNAseq data. An additional 6% are alternatively spliced but not differentially expressed in the RNAseq data. Similar to the class of differentially expressed/alternatively spliced genes found to be immunologically important in the RNAseq data, we found 5 significantly enriched ImmuneMap pathways for the class of genes that were both differentially bound in the RIPseq data and alternatively spliced in the RNAseq data (Fig 2F). The relatively small number of genes in the network belies the critical role that U2AF2 plays in regulating the alternative splicing of genes during important T cell activation processes: TCR, IL21, MAPK and NLR signaling and costimulation. These results confirm that the expression and alternative splicing changes generally observed during activation are reflected in the U2AF2 associated transcripts that are undergoing splicing.

### The activation-dependent U2AF2 interactome

**Fig 3:**
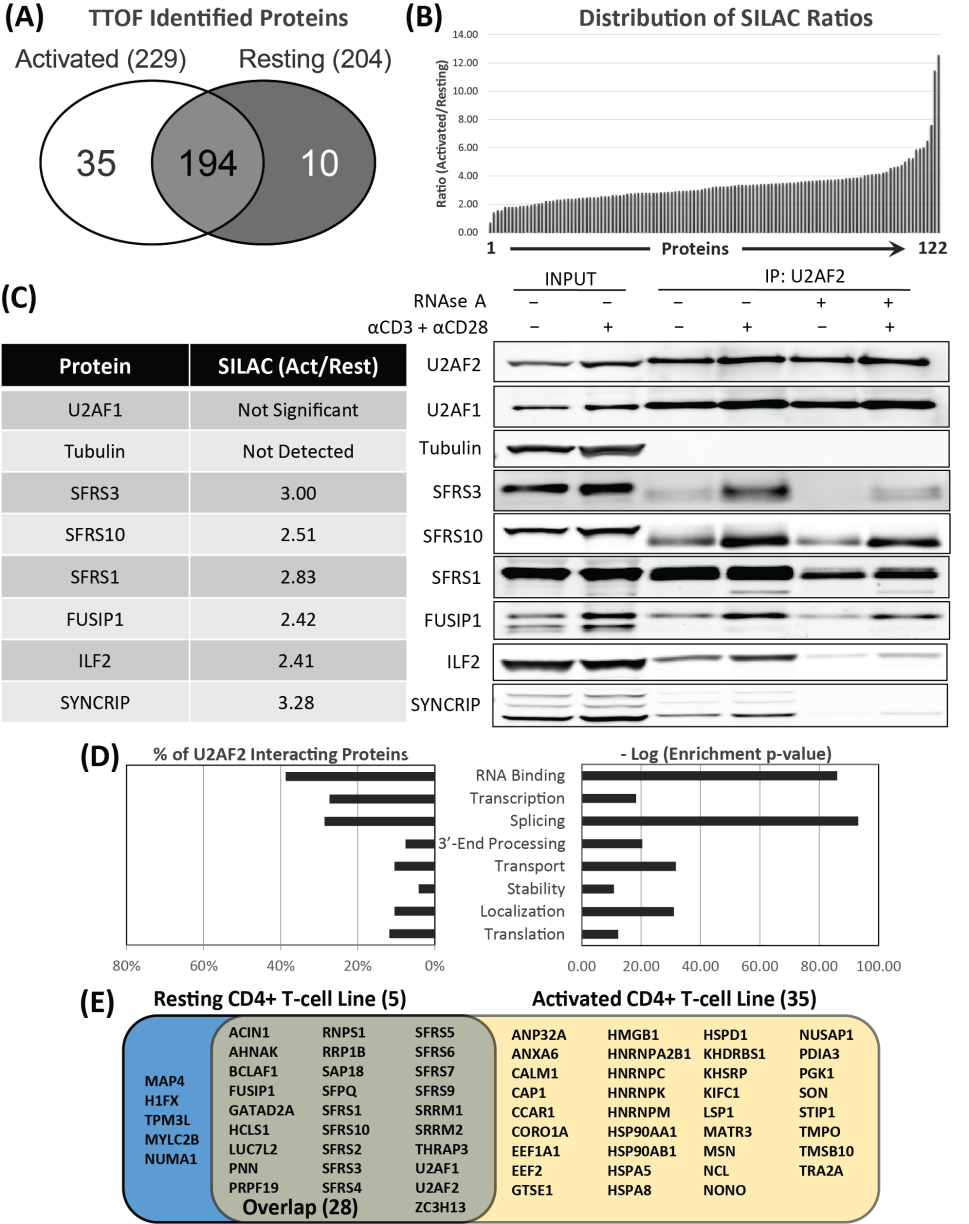
RIP-MS analysis reveals the activation-induced U2AF2-bound Core Interactome Members (CIMs) and Peripheral Interactome Members (PIMs). **(**A) Venn diagram of the complete U2AF2 interactome established by RIP-MS in resting (204 total proteins) and activated CD4 T cells (229 total proteins) with the majority shared between the two states (n = 6). (B) Mean peptide ratios for proteins detected by SILAC as significantly changed upon activation after U2AF2 IP and MS (n = 3). Almost all proteins shared between resting and activated states, increase with activation. (C) SILAC ratios (activated/resting) and Western validation of pulldowns after IP for U2AF2 in resting and activated samples with and without RNAse A treatment (representative experiment). (D) Distribution of U2AF2 interacting proteins by “RNA binding”-related GO Catergories as a percentage of the total and -log of enrichment p-value (hypergeometric test adjusted p-value <0.05). (E) Venn diagram showing the Core Interactome Members (CIMs) as defined by RNAse A treatment of U2AF2 IPs followed by proteomic mass spectrometry (n = 4/group).

The alternative splicing of particular mRNAs upon T cell activation is expected to involve association of specific regulatory factors with those transcripts. In order to identify these factors that differentially bind critical genes during T cell activation, we used mass spectrometry to identify the protein interactome of the U2AF heterodimer in resting and activated T cells. The protein component of the immunoprecipitate from the U2AF2 RIP experiment was purified, trypsin-digested, and analyzed using proteomic mass spectrometry (S6A Fig, S5 Table). Using the same protocol as for RIPseq, the samples for all mass spectrometry experiments were pre-absorbed with an isotype control mouse IgG. Moreover, we initially performed a series of control IgG pull-downs with mass spectrometry to create a list of background proteins that were combined with published list of contaminant proteins and subsequently subtracted from the proteomic datasets [37]. The total number of proteins identified in the complete interactome as associated with U2AF2 during RIP in either resting or activated samples (n = 6) are shown in Fig 3A. Of these, 35 proteins were unique to the activated state, 10 were unique to the resting state and 194 were shared in both states. Given the large number of shared proteins, quantitative mass spectrometry was performed using Stable Isotope Labeling by Amino acids in Cell culture (SILAC) to quantify the differences in protein levels upon activation (n = 3/group). The SILAC experiment identified 122 (out of the original 239 total proteins) that had significantly different abundance between resting and activated cells and all but one of the proteins increased abundance upon activation (Fig 3B, S5 Table). Western blotting for 7 interacting proteins with readily available and well-documented antibodies, all known to be RNA binding proteins, validated these observations (Fig 3C). An enrichment analysis using the Gene Ontology (GO) revealed that nearly 40% of the proteins in the interactome are known RNA Binding Proteins (RBPs) and, similarly, the two other major classes with high enrichment values are proteins involved in splicing and transcription (Fig 3D).

The members of the interactome that directly associate by binding one or more proteins comprising U2AF2 protein complexes, what we will call Core Interactome Members (CIM), were immunoprecipitated from resting or activated T cell lysates treated with RNase A prior to elution of the bound protein complexes from beads. The eluates were processed and analyzed by mass spectrometry (n = 4/group, Fig 3E) and Western blots (Fig 3C), allowing distinction between CIMs (RNAse-resistant) and Peripheral Interactome Members (PIMs; RNase-sensitive). The core U2AF2 interactome is comprised of 68 total proteins: 35 proteins are only seen in activated cells, 5 are only seen in resting cells, and 28 are shared by both states. The PIMs represented the remaining 171 proteins which is based on our previous analysis of the whole interactome.

What are the changes underlying an activation-induced interactome? While simply increasing the protein levels of U2AF2 and U2AF1 would be logical, these actually do not change with activation based on quantitative Westerns (Fig 3C). Activation-induced changes in the U2AF-centered protein interactome could also be due to increases in gene expression for the other interacting proteins. However, when the differential gene expression data was mined for the 122 U2AF2 whole interactome proteins detected by SILAC as significantly changed with activation, only 47 showed differential gene expression and 75 did not (S6B Fig). Post-translational modifications are another possible activation-induced mechanism for driving interactome assembly. Using ProteinPilot software to analyze the triple TOF mass spectrometry data obtained by U2AF2 RIP and then validating with the PhosphoSite database, 123 post-translational modifications of four types (PhosphoT, PhosphoS, Acetyl [K,R], Methyl [K,R]) were mapped to 41 proteins with the majority detected being phosphorylations (S6C Fig). This approach will not detect tyrosine phosphorylation. These results reveal a large number of post-translational changes with activation and that many of these changes may involve the loss of specific modifications. Another observation was that 7 of the 10 proteins with 3 or more modifications were RS domain-containing proteins (SRRM2, U2AF2, SFRS10, SFRS7, SFRS1, SFRS3, TRA2A; S6 Table) consistent with the known importance of RS domain phosphorylation in determining protein-protein interactions with U2AF2 [44]. In sum, these results reveal that cell activation creates a new U2AF interactome that is not only the consequence of increased gene expression, but could also result from post translational changes to the proteins.

### Biological effects of selectively knocking down U2AF2-interactome members

**Fig 4:**
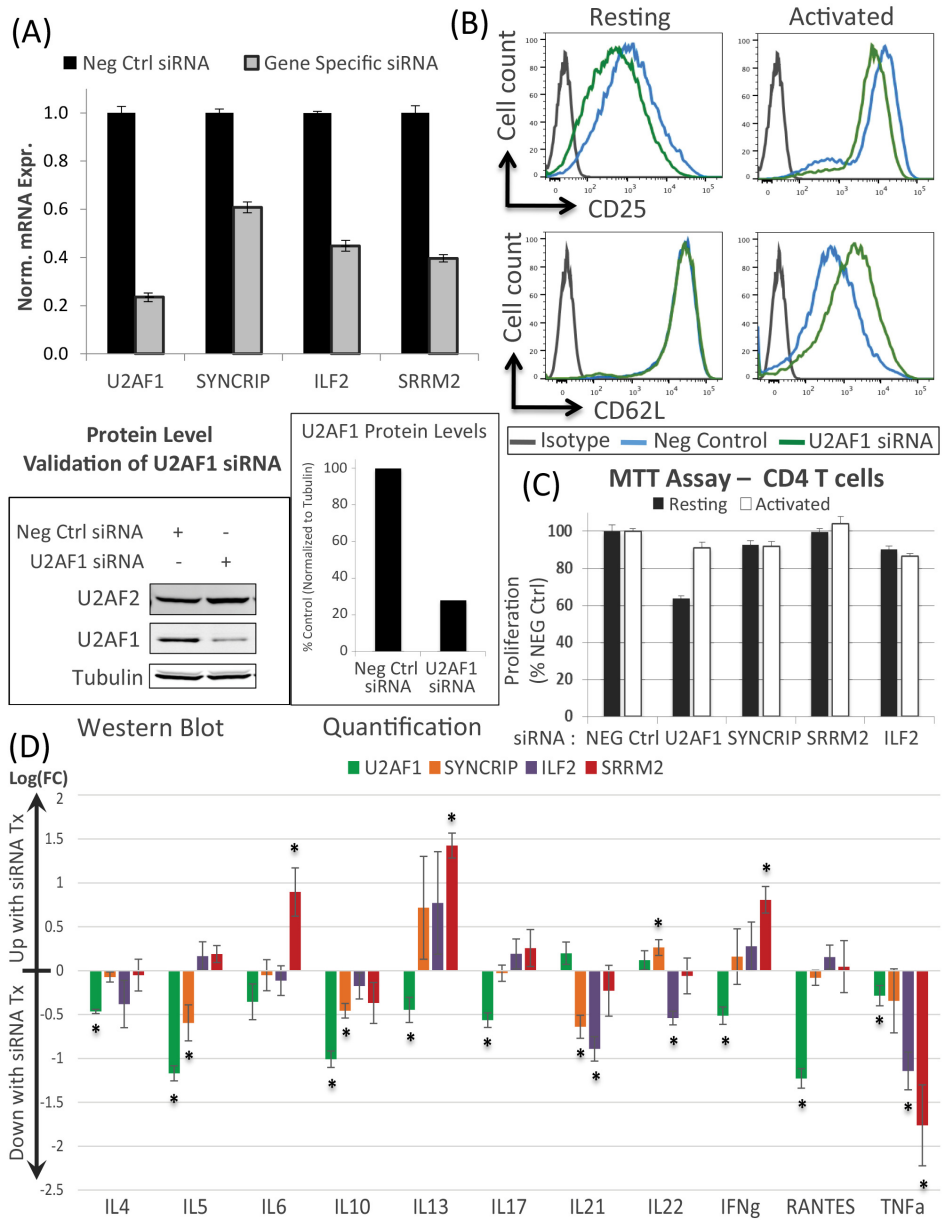
Knockdowns of U2AF2 CIMs and PIMs perturbs activation marker expression, cell proliferation and cytokine secretion. **(**A) RT-qPCR validation of siRNA knockdown for U2AF1, SYNCRIP, ILF2, and SRRM2. Error bars represent mean ± S.D. (n = 6). Immunoblot analysis shows reduced protein levels for U2AF1 with protein expression quantified and normalized to tubulin (representative experiment). (B) Histogram depicts expression of the cell activation markers CD25 and CD62L from FACS analysis of resting and activated T cells treated with negative control siRNA (blue) or U2AF1 siRNA (green). (C) Proliferation of resting and activated CD4 T cells after siRNA treatment as measured by MTT assays. Error bars represent mean ± S.D. (n = 4). (D) ELISA results for secretion of 11 cytokines after knockdown of U2AF2 interactome members plotted as log_2_ fold changes. Error bars represent mean ± S.E.M. (n = 3). Statistical significance determined by Student’s t-test (*: p <0.05).

We hypothesized that the knockdown of specific U2AF2 interactome proteins would impact the phenotypes of activated cells by affecting activation-dependent cell surface markers, cell proliferation and/or inflammatory cytokine secretion. siRNA knockdowns were done with U2AF1 and three proteins which demonstrated significantly increased binding by SILAC upon activation (Fig 4A). The four are: U2AF1 (CIM), SRRM2 (CIM), SYNCRIP (PIM) and ILF2 (PIM). Changes in surface expression of the activation markers, CD25 and CD62L, after knockdown and activation were seen only for U2AF1 (Fig 4B). Similarly, cell proliferation was reduced in the U2AF1 knockdowns of the resting cells but activation was able to override this suppression (Fig 4C). Thus, standard levels of U2AF1 expression are not required for activation-induced T cell proliferation.

Next, we used the supernatants from the siRNA treated cells to perform ELISAs on a panel of important immune cytokines. With U2AF1 knockdown (compared to siRNA control), we observed significant reduction in protein secretion of multiple critical cytokines (IL4, IL5, IL10, IL13, IL17, IFNg, RANTES, and TNFa), while knockdown of the PIMs ILF2 and SYNCRIP caused a reduction in fewer cytokines: IL21, IL22, and TNFa and IL5, IL10 and IL21, respectively (Fig 4D). Interestingly, knocking down the CIM SRRM2 increased secretion of IL6, IL13, and IFNg and decreased TNFa. The magnitude of impact on the cytokines is greater for CIMs as compared to PIMs which supports the hypothesis that proteins directly associated with U2AF2 are likely to have a larger role in determining cellular phenotype. These results, however, reveal that both core and peripheral U2AF2-interactome members can have significant, specific and different impacts on critical biological pathways associated with cytokine secretion following CD4 T cell activation.

### Changes in post-transcriptional landscapes after selectively knocking down U2AF2-interactome members

**Fig 5:**
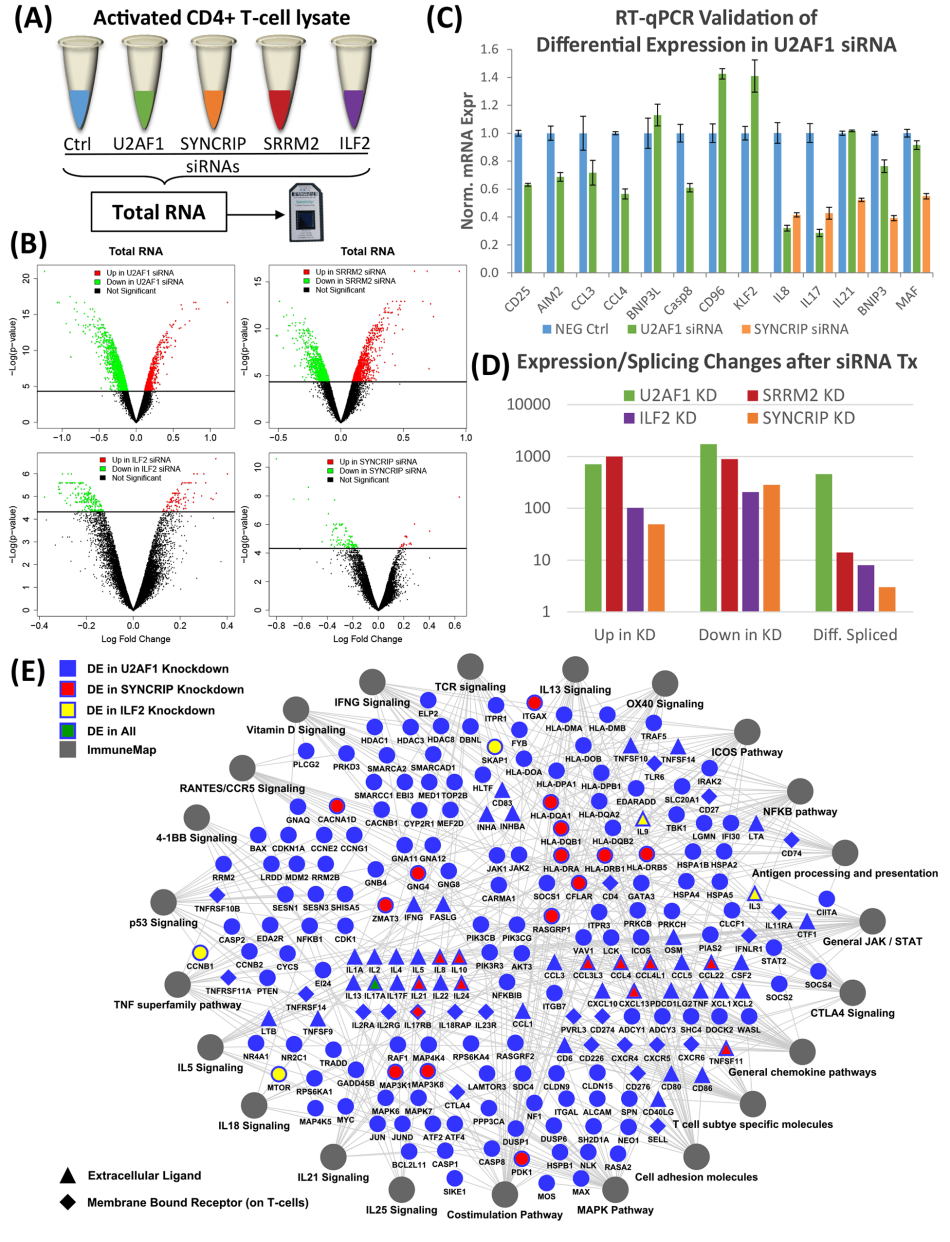
Knockdown of U2AF2 interactome members have specific impacts on the transcriptional landscape. (A) Schematic for knockdown of U2AF2 interactome members and whole exome splicing microarray analysis in activated CD4 T cell cultures. (B) Volcano plots of significantly differentially expressed genes (up in siRNA–red; down in siRNA–green) after U2AF1, SRRM2, ILF2, or SYNCRIP knockdowns. Statistical significance determined by edgeR analysis (FDR adjusted p-value <0.05, n = 3). (C) RT-qPCR validation of select differentially expressed genes in U2AF1 (green) or SYNCRIP (orange) knockdowns. Error bars represent mean ± S.D. (n = 3). (D) Total genes differentially expressed and alternatively spliced in knockdowns. Alternatively spliced genes determined by Alt-Analyze (ASPIRE > 0.2); differentially expressed genes same as (B). (E) Cytoscape map of differentially expressed genes within significantly enriched immune related pathways (hypergeometric test adjusted p-value <0.05) affected by knockdown of U2AF1 (blue). Genes from these pathways differentially expressed in ILF2 (yellow), SYNCRIP (red), or all (green) knockdowns are highlighted.

Given these significant immune phenotype changes, gene expression profiling was done after the same siRNA knockdowns. DNA microarrays were used that are specifically designed to provide both gene expression and splicing information by a combination of exome-level and exon junction probes (Fig 5A). Post-activation RNA profiles were compared between transcript-specific siRNAs and negative control siRNAs to create lists of significantly differentially expressed and/or spliced genes (S7 Table). Using normalized gene expression intensities, we generated volcano plots to show the effect of each knockdown on the expressed transcriptome (Fig 5B). The differential effects of U2AF1 and SRRM2 siRNAs (CIMs) on the global transcriptional profiles are far greater based on the number of up and down-regulated genes than the effects of knocking down ILF2 and SYNCRIP (PIMs). This result would be predicted based on our observations on the impact of CIMs vs. PIMs on immune cytokines. Additional analysis compared the overlap of the sets of differentially expressed genes affected by the knockdown of each interactome member (S7 Fig). The results show that 530 genes are impacted by both U2AF1 and SRRM2 knockdowns (CIMs), while only 18 overlap for ILF2 and SYNCRIP (PIMs). RT-qPCR of selected, differentially expressed genes validated all the candidates tested for both U2AF1 and SYNCRIP (Fig 5C). The global effects of knockdown of each protein on both expression and splicing are quantified in Fig 5D. Consistent with the critical role of the U2AF heterodimer in defining and binding to 3’ splice sites, the number of differentially spliced genes observed with U2AF1 knockdown is two orders of magnitude or more greater than seen with SRRM2, ILF2, and SYNCRIP.

To understand the significance of these global findings in immunological terms, we mapped enrichment of genes affected by the CIMs, U2AF1 and SRRM2, to specific immune pathways (Fig 5E and S8A Fig). Transcriptional changes due to U2AF1 knockdown revealed enrichment of 23 different immune pathways and SRRM2 knockdown was associated with 38 significantly enriched pathways. 18 of the pathways were unique to SRRM2, including interleukins, chemokines and multiple signaling pathways, while 20 were shared with U2AF1, including interleukins, costimulation, and different signaling pathways. U2AF1 knockdown affected the expression and splicing of 214 genes in enriched immunological pathways, the majority of which were downregulated (143/214; 67%; S8B Fig). In sharp contrast, the same analysis for SRRM2 knockdown revealed 159 immune genes, the majority of which demonstrated significantly increased expression (131/159; 82%). These results support the novel conclusion that, in comparison to U2AF1, SRRM2 functions for many genes as a post-transcriptional inhibitor during T cell activation. That conclusion is consistent with the marked increase in cytokine secretion observed with SRRM2 knockdown discussed above.

We also compared genes affected by U2AF1 knockdown to those differentially expressed with knockdown of the PIMs, ILF2 or SYNCRIP (Fig 5E). Knocking down SYNCRIP or ILF2 influenced the activation-induced expression of far fewer genes (123 and 308, respectively) though these were still fundamentally important genes to CD4 T cell functions. Specifically, expression of critical cytokines and secreted molecules (IL17, IL21, IL8, IL10, CCL4, TNFSF11) decreased with SYNCRIP knockdown. ILF2 knockdown decreased expression of IL3, IL9, mTOR, and CCNB1 but increased expression of IL17 and SKAP1. In sum, we observed three classes of organizational topology with knockdown of U2AF2 interactome members: one protein can impact more genes but fewer pathways (U2AF1), fewer genes but more pathways (SRRM2), or relatively few genes and pathways (ILF2 and SYNCRIP) but ones with great biological significance for T cell activation.

**Fig 6:**
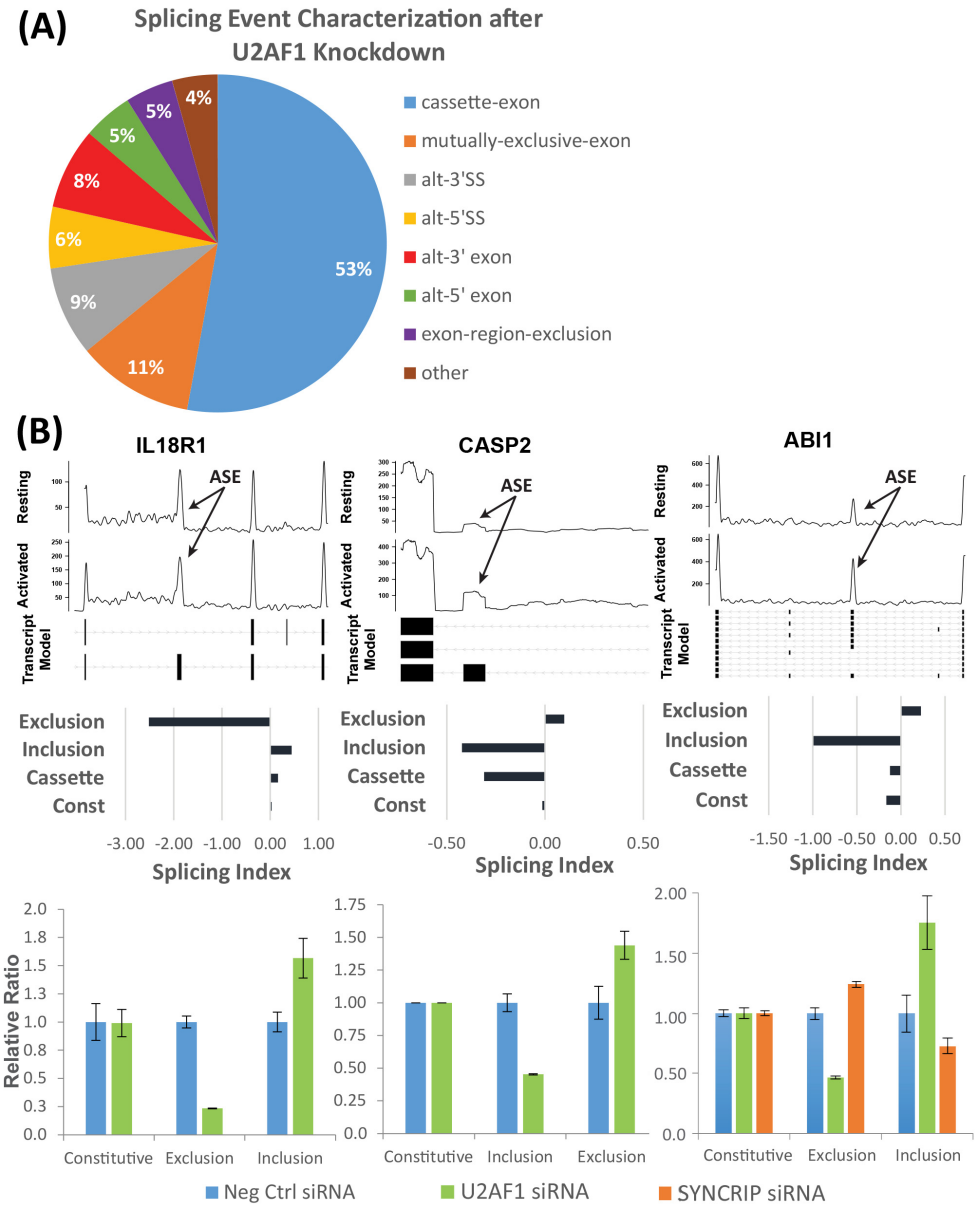
U2AF1 knockdown in activated T cells causes significant splicing changes. (A) Breakdown of differential splicing events affected by knockdown of U2AF1 in whole exon splicing microarray data using Alt-Analyze. (B) *Top*: RNAseq coverage plot of regions differentially spliced between resting and activated T cells for IL18R1, CASP2, ABI1. *Middle*: Normalized microarray intensities for junction probes (Exclusion/Inclusion) and exon probes (Cassette and Constitutive) from Control and U2AF1 siRNA samples are used by the Transcript Analysis Console (Affymetrix) to calculate the Splicing Index (SI). Greater probeset level intensity values in the Control siRNA samples correspond to negative SI values and positive SI represents increased intensity in the U2AF1 siRNA samples. *Bottom*: RT-qPCR Validation of alternative splicing after U2AF1 (green) or SYNCRIP (orange) knockdowns. ASE–Alternatively Spliced Exon. Error bars represent mean ± S.D. (n = 3).

The same microarray data was used to analyze the changes in alternative splicing created by knockdowns of these 4 RNA-binding proteins using the program Alt-Analyze. The most common type of splicing event for the U2AF1 knockdown involves cassette exons (Fig 6A) and this is also true at the global level by paired-end RNAseq for resting vs. activated CD4 T cells (Fig 1C). Differential splicing of mutually exclusive exons is the second most common impact of U2AF1 knockdown. Importantly, this class of splicing event is not seen in RNAseq data for untreated CD4 T cells, resting vs. activated. At the isoform level, the two most common assignable switching events after U2AF1 knockdown are alternative C-terminal (29%) and N-terminal (28%) variants. These results are also consistent with the RNAseq data as well as a previous study in HEK293 cells that assessed the impact of U2AF1 knockdown on alternative splicing events [45].

Three targets were validated from the list of alternatively spliced genes in the U2AF1 knockdown that were also alternatively spliced upon activation (Fig 6B). Several novel results were documented. First is the increased exclusion for IL18R1 of a specific cassette exon with activation. In contrast, U2AF1 knockdown specifically prevents this splicing event. Second, for CASP2 and ABI1, there is increased exon inclusion upon T cell activation that is reversed by U2AF1 knockdown. On the other hand, SYNCRIP knockdown results in the opposite effect with an amplification of the natural activation-induced alternative splicing event of exon inclusion in ABI1.

### U2AF1 regulates activation-induced assembly of U2AF2 protein/RNA complexes

**Fig 7:**
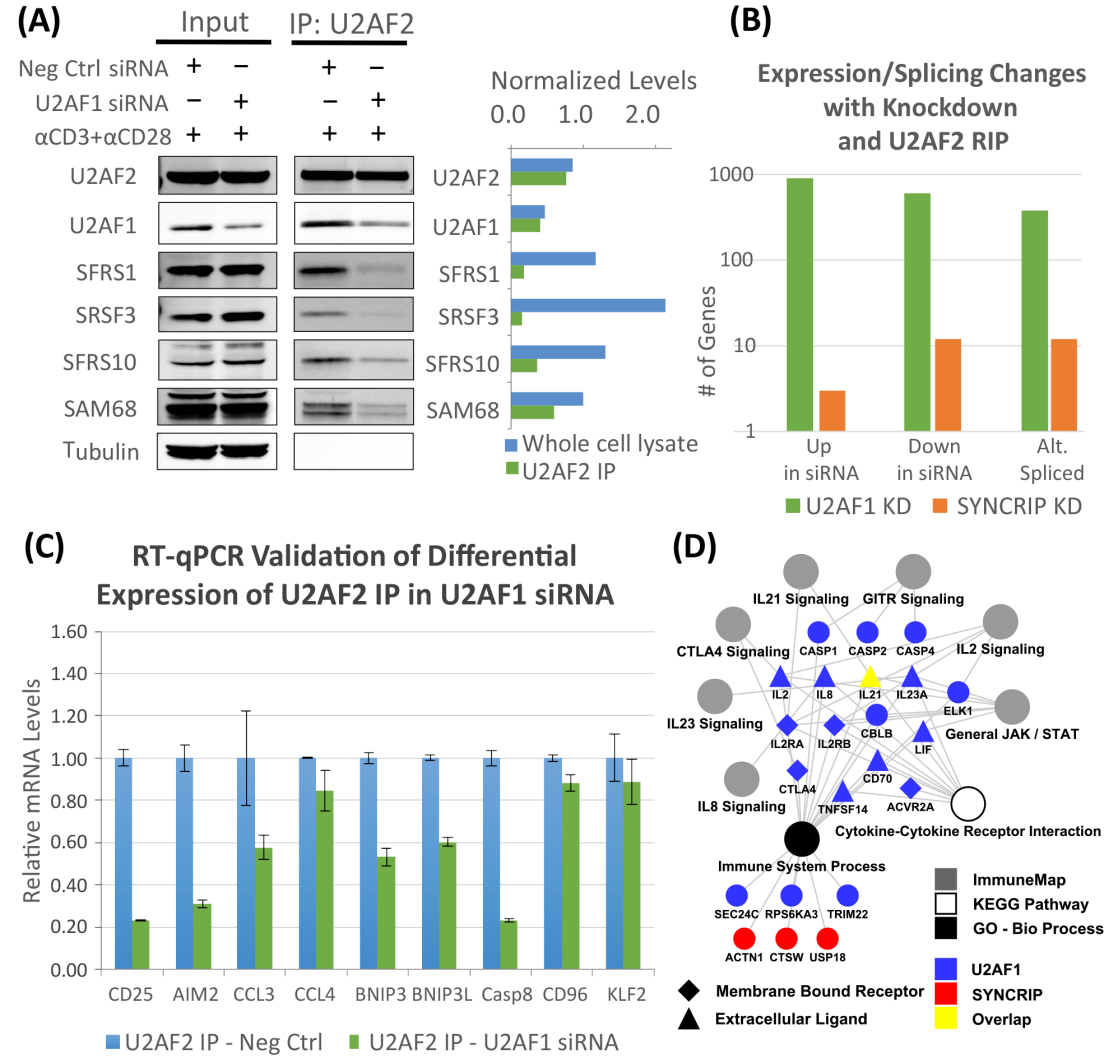
U2AF1 knockdown impairs protein/RNA interactions with U2AF2 in activated T cells. (A) Immunoblot analysis for binding of select U2AF2 interactome members after immunoprecipitation with U2AF2 antibody in activated cells with negative controls vs. U2AF1 siRNA. Protein expression quantified using the Li-Cor Odyssey and normalized to tubulin. (B) Totals of genes differentially bound to U2AF2 after knockdown of U2AF1 and SYNCRIP as compared to negative control siRNAs. Statistical significance determined by edgeR analysis (FDR adjusted p-value <0.05, n = 3). (C) RT-qPCR validation of selected differentially expressed genes after U2AF1 knockdown as revealed by U2AF2 RIP. Error bars represent mean ± S.D. (n = 3). (D) Cytoscape map of genes differentially bound to U2AF2 within KEGG pathways, Gene Ontology, and ImmuneMap after knockdown of U2AF1 (blue) or SYNCRIP (red). Statistical significance determined by hypergeometric test (multiple testing corrected p-value <0.05).

U2AF2 differentially binds critical genes that are differentially expressed and/or alternatively spliced during T cell activation. The data also confirm that members of activation-induced U2AF2 protein complexes affect post-transcriptional regulation of genes key to T cell activation. To merge these concepts into one type of experiment, selected U2AF2 interactome members were knocked down with siRNA followed by IP for U2AF2 and probing for different U2AF2 interacting partners by Western blots. U2AF1 knockdown in activated CD4 T cells results in a dramatic decrease in binding for all tested proteins relative to the negative control siRNA (Fig 7A). These results demonstrate that U2AF1 is critical to the integrity of U2AF2 centered protein-protein complexes assembled with activation and not simply mediating binding to RNA via 3’ splice sites. In contrast, knockdown of SYNCRIP, ILF2, and SRRM2 did not prevent recruitment of the proteins tested by Westerns to the U2AF2/U2AF1 splicing complex (S9 Fig). Thus, the varied impacts of knocking down SYNCRIP, ILF2, and SRRM2 are not the result of impairing assembly of the interacting proteins tested in these Westerns.

### U2AF2 RIPchip reveals the transcripts bound and the expression regulated by interaction with U2AF1 and SYNCRIP

To directly identify the impact of core vs. peripheral interactome members on the RNA-binding profile of U2AF2 after 24 hours of activation, RIPchip was performed following knockdowns of U2AF1 or SYNCRIP. RIPchip using exon/junction microarrays provides the identity of the bound transcripts, measures expression levels and reveals distinct isoforms resulting from alternative splicing following knockdowns of one CIM (U2AF1) and one PIM (SYNCRIP). The U2AF2 RIPchip at 24 hours identified 1814 unique genes whose expression changed after T cell activation when U2AF1 is knocked down (906 up-regulated, 604 down-regulated and 379 alternatively spliced; Fig 7B). There were 27 unique genes changed with SYNCRIP knockdown (3 up-regulated, 12 down-regulated and 12 alternatively spliced; S8 Table). The number of genes differentially bound to U2AF2 following the knockdowns compared to siRNA controls is more than two orders of magnitude greater for the CIM as compared to the PIM. Only 8 of the 27 SYNCRIP transcripts were also impacted by U2AF1 knockdown, including the Tfh-defining cytokine, IL21. For the U2AF1 data set, we validated differential expression with the same 9 immunologically important genes as previously done for U2AF2 RIPseq (Fig 2D). Consistent with the role of U2AF1 in facilitating RNA binding by U2AF2, 6 of the 9 genes that increase expression with activation significantly decreased when U2AF1 was knocked down (Fig 7C). Finally, U2AF2-bound gene expression changes linked to U2AF1 or SYNCRIP knockdowns were mapped to enriched ImmuneMap and KEGG pathways and GO categories. Seven immune pathways including IL2, IL8, IL21, IL23, GITR, CTLA4, and JAK/STAT signaling were identified as significantly enriched (Fig 7D). Thus, using RIPchip to identify U2AF2 bound genes affected by U2AF1 and SYNCRIP knockdown exemplifies the class of network topology in which regulation of a few genes is linked to a small but critically important set of immune pathways. A PIM like SYNCRIP has a limited effect on U2AF2’s ability to bind transcripts compared to U2AF1, yet the transcripts affected by SYNCRIP in the context of their interaction with U2AF2 still map to immune pathways that are important for T cell activation. These results support the hypothesis that both CIMs and PIMs function to determine the activation-induced T cell transcriptome, but do so within a relative hierarchy to differentially regulate assembly of the RNA and protein components of the U2AF2 interactome.

## Discussion

Understanding the complexities of T cell activation in human health and disease requires studies of primary human cells though significant advances have been made using cell lines. We developed a scalable model of T cell activation by activating primary CD4 T cells isolated from normal human blood donors and culturing them in the presence of rIL2. These cells displayed a distinct cellular phenotype upon activation that included canonical markers for T cell activation and cytokine secretion. At the transcriptional level, we observed large numbers of differentially expressed and alternatively spliced genes following activation, consistent with the expected behavior of primary cells [46, 47]. The presence of a class of genes that were both differentially expressed and alternatively spliced was also confirmed and these genes are important for the proliferation and differentiation of the cells as well as key immune functions.

We hypothesized that splicing complexes are critical post-transcriptional regulators of activation-induced differential gene expression and alternative splicing. The RNA interactome of the central splicing complex member U2AF2 was identified in human CD4 T cells using RIPseq and revealed that the U2AF2-bound mRNA transcripts detected represent 82% of the transcripts expressed at the total RNA level. The genes differentially bound to U2AF2 were associated with a large network of immune pathways and the subset of differentially bound, alternatively spliced genes was significantly enriched for immune pathways such as CD28 costimulation, T cell receptor, MAPK, and IL21 signaling. Thus, the RIPseq data provides evidence for the critical role of U2AF2 in regulating the transcriptome of activated human primary CD4 T cells.

The next goal was to identify the protein interactome of U2AF2 to address the regulation of CD4 T cell activation by assembly of RNA binding protein complexes. One definition of an interactome is all the proteins that directly bind and interact with the target protein. For our studies, this is determined by RNAse-resistant interactions that imply the identified protein is associated with the U2AF2 multi-protein complex. However, because we are working with RNA-binding proteins, there is another layer of biologically significant information that can be captured by analyzing all the proteins that indirectly associate with U2AF2 through the larger context of the RNA scaffold. This is also consistent with the idea that there is a high level of three-dimensional RNA structure that could facilitate the functional regulation of the U2AF complex by RNA binding proteins that are RNAse-sensitive and located some distance away [48, 49]. Specifically, RNA binding proteins that are located at some distance from the U2AF complexes assembled might function to block U2AF recruitment to their binding sites. For example, one of the RNAse sensitive proteins identified by our mass spectrometry proteomics is hnRNPA1 which has been shown to bind RNA at specific regions to suppress splicing [50].

Therefore, we refer to RNase-resistant U2AF2 interacting proteins as the Core Interactome Members (CIMs) while also referring to a second layer of interactome proteins that are assembled on the same transcripts but different locations as Peripheral Interactome Members (PIMs). Using RIP without RNAse treatment followed by mass spectrometry showed that U2AF2 associates with 239 proteins in resting or activated cells. The CIM subset comprised 68 proteins and we classified the additional 171 proteins as PIMs. These PIMs have their own direct interacting proteins that are being assembled in an activation-dependent manner in CD4 T cells and our results demonstrate that knocking down PIMs have significant impacts on gene expression and cytokine secretion. Moreover, when we knock down the PIM SYNCRIP prior to U2AF2 RIPchip, we demonstrate a small but important number of changes in U2AF2’s ability to bind transcripts, consistent with the argument that PIMs regulate the transcriptome in a U2AF2-dependent manner.

Quantitative experiments using U2AF2 RIP followed by SILAC mass spectrometry showed that nearly all of the detectable interactome member proteins shared between activated and resting states significantly increase in the activated cells despite a minimal activation-induced change in the level of U2AF2 protein. These increased associations are not completely explained by activation-induced changes in transcript expression of the interactome proteins because only 38.5% of the proteins with observed SILAC differences (47/122) had correlated changes at the RNA level based on the RNAseq data. One potential mechanism to explain the increased association of PIMs with U2AF2 would be through activation-dependent changes in transcript abundance on which both proteins were bound. Finally, post-translational modifications are another canonical mechanism of regulating the assembly of protein complexes. Indeed, we identified 41 proteins in the complete interactome (17%) with differential post-translational modifications upon activation. RS domain-containing proteins (including U2AF2) comprised 7 of the top 10 post-translationally modified proteins and phosphorylation was the most common modification. Supporting these results in our RNAseq data is the activation-induced increase of the transcript for SR Protein Kinase 1 (SRPK1, S1 Table), which is known to phosphorylate the RS domains of SR proteins [51, 52]. It has also been shown that phosphorylation of SR proteins by SRPKs is critical for their translocation to the nucleus and spliceosome assembly at defined exons [53]. The activation-induced reorganization of the U2AF2 interactome studied specifically in CD4 T cells is consistent with our premise that an understanding of the dynamics of RNA-binding protein interactome assembly is critical to understanding T cell biology.

After compiling the U2AF2 interactome, we used siRNA knockdown of both core and peripheral U2AF2 interactome members to assess the biological impacts on T cell activation. The first step was to knock down U2AF1 (CIM). Significant phenotypic effects on the T cells included reduced cell proliferation at rest, changes in expression of activation markers, and significantly diminished secretion of 7 highly immunologically relevant cytokines. We expected a significant impact of knocking down U2AF1 because it is the U2AF2’s binding heterodimeric partner; furthermore, these results provided a benchmark for our approach to study the differential impacts of additional interactome members.

Knockdown of SRRM2 (CIM), SYNCRIP (PIM), and ILF2 (PIM) did not impact cell proliferation or activation marker expression but still significantly changed the secretion of multiple cytokines. Knockdown of SYNCRIP or ILF2 reduced IL21 secretion and are examples of target specificity and the potentially overlapping and coordinated roles of these splicing factors in an important T cell specific pathway. IL21, a hallmark of activated CD4 T cells and the defining cytokine for T follicular helper cells (Tfh), has a role in proliferation and its excessive production has been implicated in chronic inflammatory disorders like rheumatoid arthritis and psoriasis as well as graft rejection following organ transplantation [54–56]. Unexpectedly, the knockdown of SRRM2 resulted in increased secretion of 3 pro-inflammatory cytokines (IL6, IL13, IFNg) suggesting it may play an inhibitory role in post-transcriptional regulation of these critical factors. These data documenting specific changes in secreted, T cell activation-induced cytokine levels after knockdown of splicing interactome partners are the first direct demonstrations of the connection between RNA-binding proteins and immunologically significant cellular changes downstream of changes in mRNA expression and splicing. Moreover, many of the impacted cytokines are well established signals driving and/or associated with CD4 T cell subset differentiation [57–59].

The next step to investigating the biological significance of the U2AF2 interactome was to assess the transcriptional effects of knocking down the same set of interactome members. In the first series of experiments, total RNA was profiled by exon/junction microarrays at 24 hours to reveal the cumulative impact of these interacting proteins over this critical early time period in CD4 T cell activation. At the global level, given the limited cellular phenotypic impacts of SRRM2 knockdown on cell proliferation and surface activation markers, it was surprising that the differential expression of thousands of genes was affected, a similar number of genes to U2AF1. A subsequent functional enrichment analysis of these lists of differentially expressed genes identified markedly different profiles of significant immune pathways and associated genes. The network of significantly enriched immune pathways with associated genes affected by SRRM2 siRNA (S8A Fig) has fewer genes than U2AF1 (159 vs. 214) but is connected to a larger number of categories and pathways (38 vs. 23). SRRM2 targets more networks but fewer genes, consistent with the conclusion that it has evolved to regulate members common to multiple pathways. Both CIMs tested have a discrete and definable set of targeted genes that they regulate during T cell activation.

Relative to the CIMs, far fewer transcriptional changes were detected following knockdown of the PIMs, ILF2 and SYNCRIP. This, however, does not diminish the importance of the affected genes. Specifically, we observed SYNCRIP and/or ILF2 impacted expression of IL17, IL21, and FOXP3, important genes that define three of the canonical CD4 T cells subsets that emerge from T cell activation: Th17, Tfh, and Tregs, respectively. Interestingly, ILF2 knockdown decreased secretion of IL21, IL22 and TNFa at the protein level but that was not evident at the transcript level. These results are consistent with additional functions known for RNA binding proteins that do not involve direct mRNA splicing events such as regulating ribosomal entry and translation. In fact, ILF2 has been shown to regulate translation as an IRES-dependent trans-activator [60, 61].

U2AF2 RIP after knockdown of U2AF1 allows us to study the direct effects of preventing assembly of the U2AF heterodimer. At the protein level, we used western blots to confirm the critical role of U2AF1 in assembling activation-dependent protein complexes centered on U2AF2. This effect was not observed with knockdown of any other U2AF2 interactome member. While U2AF1 knockdown had a substantial global impact on the RNA interactome of U2AF2, the immune pathways containing genes affected by U2AF1 knockdown were specifically associated with various aspects of T cell activation (Fig 7D). SYNCRIP knockdown impacted IL21, IL5 and IL10 cytokine secretion and specifically reduced binding of U2AF2 to IL21 transcripts as well as changed binding to 14 other target genes. Clearly, the number of transcripts that are impacted by knocking down a CIM like U2AF1 is much larger than the impact of a PIM like SYNCRIP. However, knocking down a PIM, that is by definition a protein not directly bound to the U2AF2 protein complex, still impacts localization of U2AF2 to a number of important transcripts.

## Conclusions

**Fig 8:**
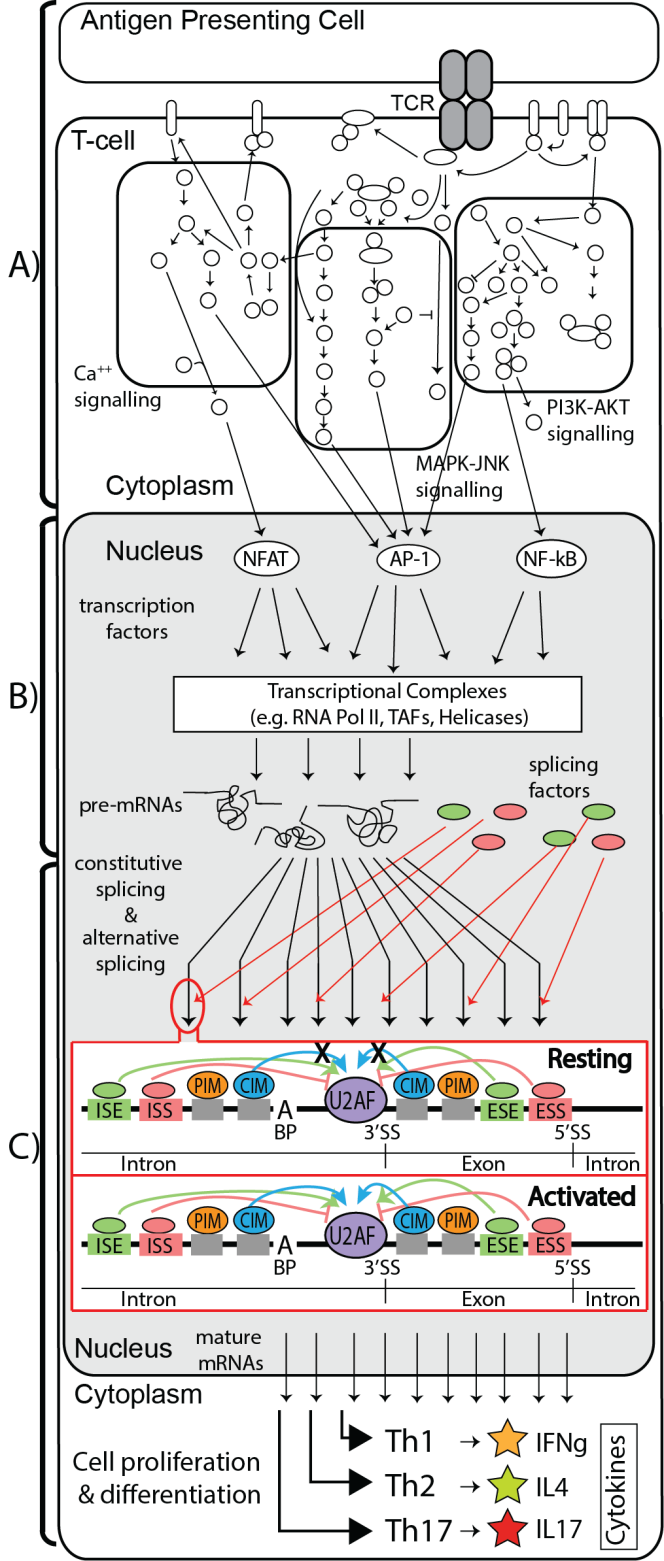
CD4 T cell activation and regulation of splicing by assembly of RNA binding protein complexes. (A) T cell activation by antigen presentation and costimulation followed by induction of T cell specific intracellular signaling pathways. (B) Activation and translocation of transcription factors to the nucleus is followed by modular assembly of transcription factor complexes and initiation of transcription. (C) Assembly of U2AF centered RNA-protein-protein complexes mediates post-transcriptional regulation, including canonical and alternative splicing, of specific transcripts resulting in an appropriate activation-induced T cell response. *Red box*: A view of an individual pre-mRNA transcript, in Resting and Activated T cells, adds CIMs and PIMs to the current model for recruitment of the U2AF heterodimer to the 3’SS via splicing enhancers and suppressors that determine and regulate U2AF2 mediated canonical and alternative splicing. BP–branch point; ESE–exonic splicing enhancer; ESS–exonic splicing suppressor; ISE–intronic splicing enhancer; ISS–intronic splicing suppressor.

Many current paradigms in immunology for transcriptional regulation and understanding the construction of a T cell’s transcriptome have focused on the upstream roles of TCR and costimulatory signaling to activate the function of various transcription factors and their partners (Fig 8A). This work has been critically important to defining the nature of T cell immunity. While transcription determines and drives the original set of pre-mRNAs (Fig 8B), our work underlines the importance of another level of regulation between transcription and translation that involves both canonical and alternative splicing and is equally critical for understanding the CD4 T cell immune response in molecular terms. Indeed, this mechanism of regulating T cell activation can be considered in the context of the growing appreciation of regulating cellular responses by epigenetic modifications, miRNAs and long non-coding RNAs. We propose that T cell activation drives assembly of RNA-protein-protein complexes on individual transcripts and these complexes regulate the splicing events and ultimately the expression of these genes at both the transcript and protein levels (Fig 8C). There are only a few examples of the intersection of the immunology and splicing fields that are really well studied at the molecular level; for example, CD45 splicing is regulated by specific RNA binding proteins such as hnRNPL, hnRNPLL, hnRNPA1 and SFPQ that cooperate in aligning intron/exon junctions or regulate the recruitment of the spliceosome [62–65]. All four of these proteins were identified in our U2AF2 interactome.

Our results support a model in which activation leads to the modular assembly of two classes of multi-protein RNA binding complexes (CIMs and PIMs) that determine the nature of activation-induced regulation of gene expression in primary human CD4 T cells. This work demonstrates the importance of RNA binding proteins in actively contributing to the regulation of T cell activation and function. However, while this model is necessarily focused on immunity, the general rules of activation-induced interactome assembly and regulation of both canonical and alternative splicing are also likely to be relevant to many different cell activation agendas that are central to understanding changes in health and disease.

## Supporting Information

S1 Fig Phenotypic and activation markers in Jurkat T cells. (A) ELISA results for secretion of 12 cytokines in Jurkat T cell culture. (B) Histogram depicts expression of the T cell marker CD4 and various activation markers (CD25, CD69, CD71, CD62L, and CD40L) from FACS analysis of resting (red) and activated (blue) Jurkat T cell culture.
(PDF)

S2 Fig The CD4 T cell culture displays large changes in expression and splicing upon activation. (A) Heatmap of fpkms for 6,382 significantly differentially expressed genes in the resting and activated CD4 T cell culture. (B) RT-qPCR validation of alternative splicing for 15 genes with differential expression of cassette exons as determined by Alt-Analyze.
(PDF)

S3 Fig Examples of activation-induced alternative splicing in CD4 T cells. Sashimi plots demonstrate alternative splicing events that result in isoforms with alternative N-termini. Activation causes reduced expression of the minor isoform in RERE (A) and RTN4 (B) while increasing expression of the minor isoform in CCM2 (C). Orange circles highlight regions increased in activated samples relative to resting and green circles highlight the opposite.
(PDF)

S4 Fig Examples of activation-induced alternative splicing in CD4 T cells. Sashimi plots demonstrate alternative splicing events that result in isoforms with alternative C-termini. Activation causes increased expression of the minor isoform in CEP63 (A). In PREB (B) and PXN (C), activation causes increased inclusion of a cassette exon containing a stop site. Orange circles highlight regions increased in activated samples relative to resting and green circles highlight the opposite.
(PDF)

S5 Fig U2AF2 RIPseq identifies the RNA transcripts bound by U2AF2. (A) Schematic of the U2AF2 RIP experiment followed by RNAseq. (B) Hexbin plot of U2AF2 RIPseq log_2_ fold changes (resting vs. activated) on the x-axis versus RNAseq log_2_ fold changes (resting vs. activated) on the y-axis shows that the methods have comparable results on a per gene basis.
(PDF)

S6 Fig RNA expression changes of U2AF2 interacting proteins and the breakdown of post-translational modifications. (A) Schematic of the U2AF2 RIP experiment followed by tripleTOF mass spectrometry. (B) Breakdown of the differential expression and splicing changes for the transcripts of U2AF2 interacting proteins. (C) Distribution of detected post-translational modifications in resting and activated cells after U2AF2 RIP-MS and the breakdown by type of modification in each group.
(PDF)

S7 Fig Knockdown of U2AF2 interacting proteins in activated T cells has a variable effect on differential gene expression. Overlap of differentially expressed genes in activated T cells with knockdown of U2AF1, SRRM2, SYNCRIP, and ILF2.
(PDF)

S8 Fig ImmuneMap pathway enrichment for genes differentially expressed with CIM knockdown. A Cytoscape network of enriched immune pathways (gray) containing differentially expressed genes (downregulated–blue, downregulated—red) in activated T cells with (A) SRRM2 and (B) U2AF1 knockdown.
(PDF)

S9 Fig Effects of knocking down U2AF2-interacting partners on the assembly of the U2AF2 protein complex. Immunoblot analysis for binding of select U2AF2 Interacting Proteins after immunoprecipitation with U2AF2 antibody in activated Jurkat T cell samples treated with the specified siRNA.
(PDF)

S1 Table List of differentially expressed or alternatively spliced genes upon activation of CD4 T cells. (XLSX)

S2 Table Genes that are differentially expressed and alternatively spliced are enriched for GO categories and immune pathways. Statistics for enrichment of Gene Ontology–Biological Process categories (p-value < 0.05) of differentially expressed and alternatively spliced genes.
(PDF)

S3 Table Genes that are differentially expressed and alternatively spliced are enriched for GO categories and immune pathways. Statistics for enrichment of ImmuneMap pathways (adjusted p-value < 0.1) of differentially expressed and alternatively spliced genes.
(PDF)

S4 Table List of genes differentially bound to U2AF2 upon activation of CD4 T cells. (XLSX)

S5 Table List of proteins bound to U2AF2 as determined by mass spectrometry. (XLSX)

S6 Table Top 10 U2AF2 interactome members with detected post-translational modifications. Distribution of post-translational modifications in resting and activated T cells and the breakdown by type of modification for each protein.
(PDF)

S7 Table List of genes differentially expressed or alternatively spliced upon siRNA knockdown of U2AF2 interactome members. (XLSX)

S8 Table List of genes differentially bound to U2AF2 upon siRNA knockdown of U2AF1 and SYNCRIP. (XLSX)
