# Supplementary figures and images for: The Activation-Induced Assembly of an RNA/Protein Interactome Centered on the Splicing Factor U2AF2 Regulates Gene Expression in Human CD4 T Cells
Source: PLoS One. 2015 Dec 7;10(12):e0144409. doi: 10.1371/journal.pone.0144409 (PMC4671683; doi:10.1371/journal.pone.0144409)

**(A)**

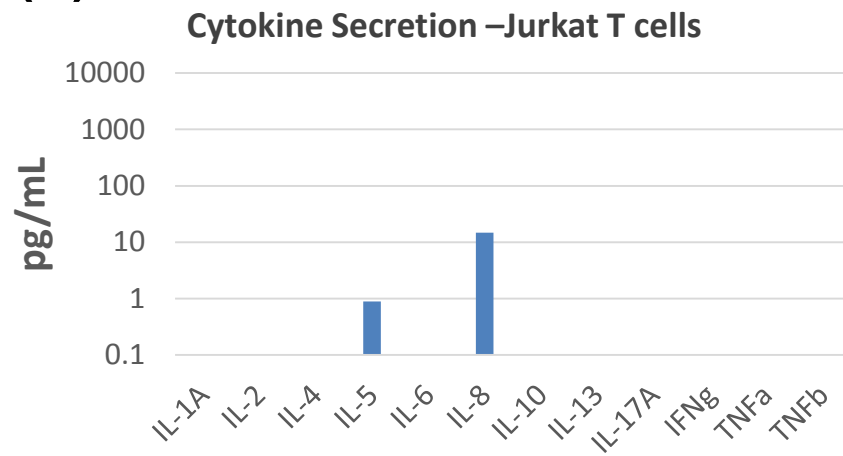

**(B)**

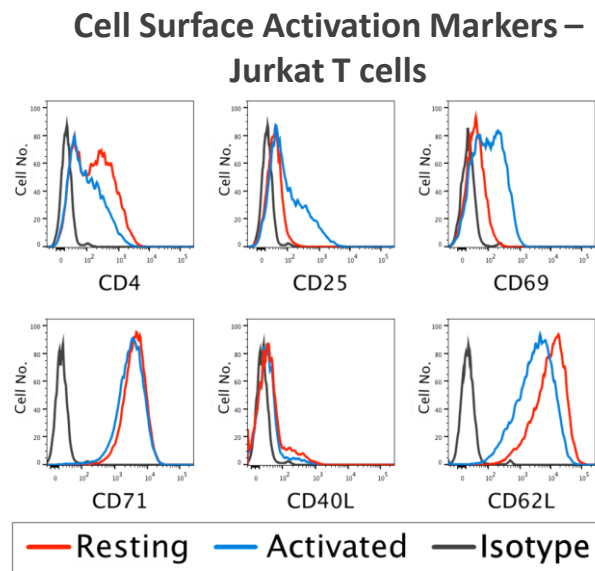

Supplement: S1 Fig — (A) ELISA results for secretion of 12 cytokines in Jurkat T cell culture. (B) Histogram depicts expression of the T cell marker CD4 and various activation markers (CD25, CD69, CD71, CD62L, and CD40L) from FACS analysis of resting (red) and activated (blue) Jurkat T cell culture. (PDF) [file pone.0144409.s001.pdf]

## (A) Differentially Expressed Genes

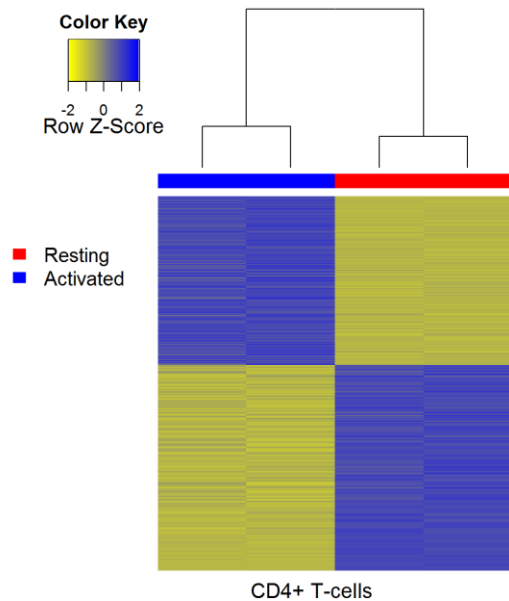

## (B) Alt Splicing Validation by RT-qPCR

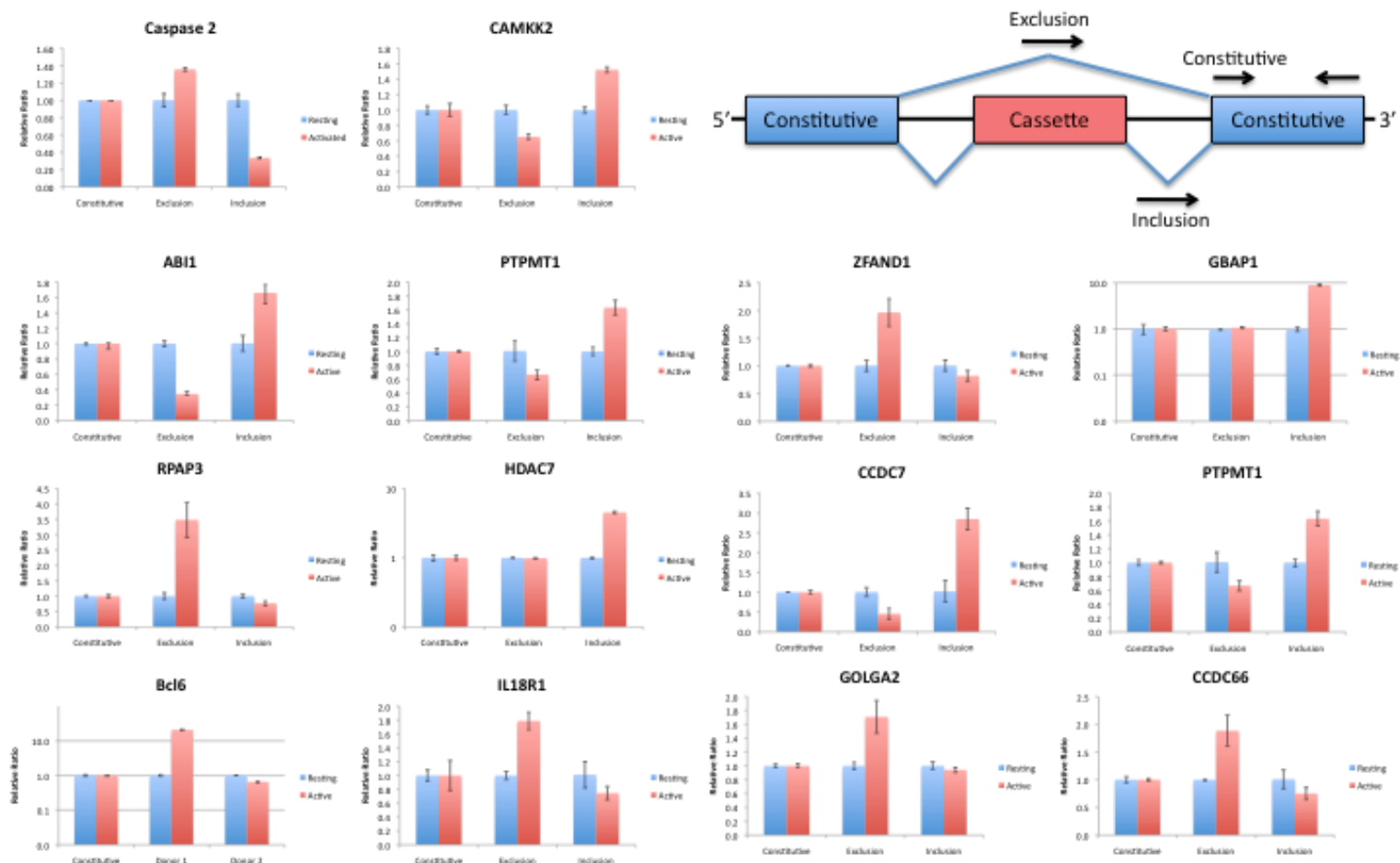

Supplement: S2 Fig — (A) Heatmap of fpkms for 6,382 significantly differentially expressed genes in the resting and activated CD4 T cell culture. (B) RT-qPCR validation of alternative splicing for 15 genes with differential expression of cassette exons as determined by Alt-Analyze. (PDF) [file pone.0144409.s002.pdf]

## Alternative N-terminus    — Resting    — Activated

### (A) RERE

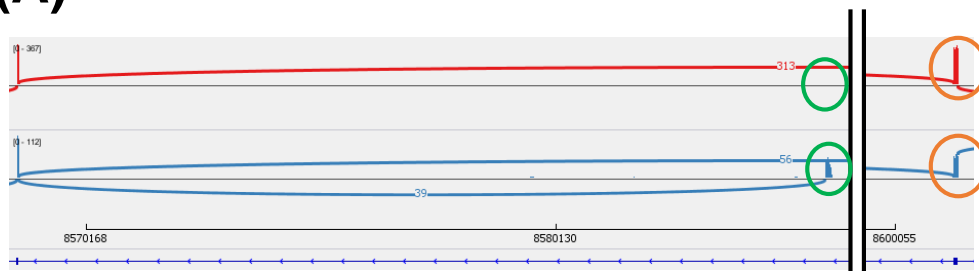

### (B) RTN4

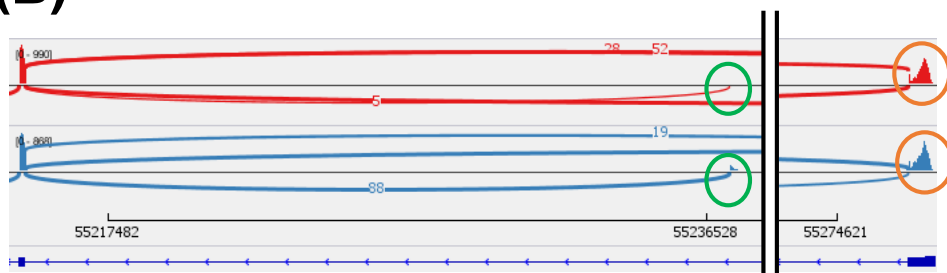

### (C) CCM2

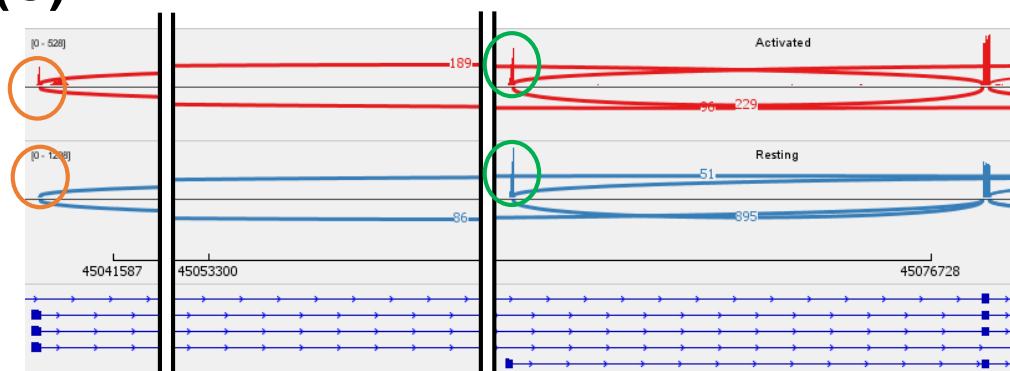

Supplement: S3 Fig — Sashimi plots demonstrate alternative splicing events that result in isoforms with alternative N-termini. Activation causes reduced expression of the minor isoform in RERE (A) and RTN4 (B) while increasing expression of the minor isoform in CCM2 (C). Orange circles highlight regions increased in activated samples relative to resting and green circles highlight the opposite. (PDF) [file pone.0144409.s003.pdf]

# Alternative C-terminus    — Resting    — Activated

## (A) CEP63

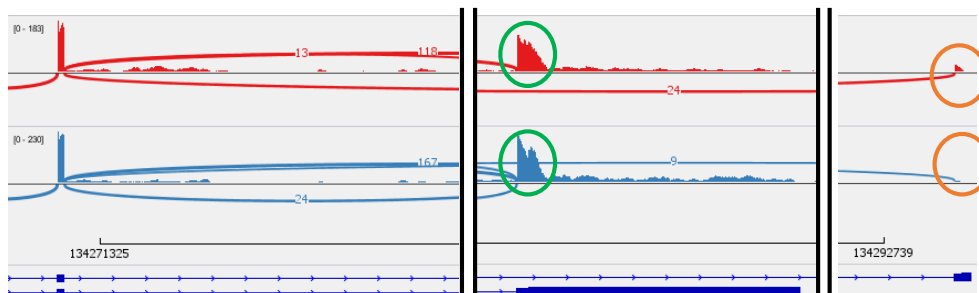

## (B) PREB

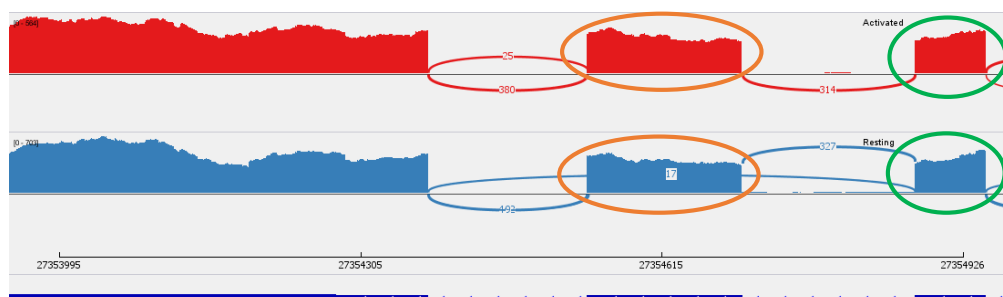

## (C) PXN

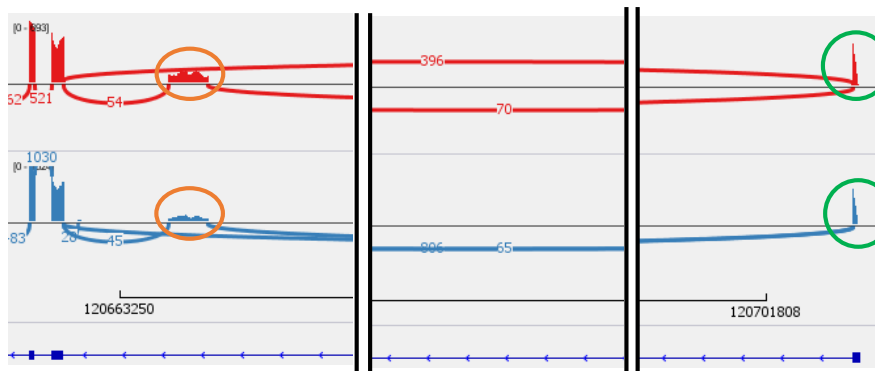

Supplement: S4 Fig — Sashimi plots demonstrate alternative splicing events that result in isoforms with alternative C-termini. Activation causes increased expression of the minor isoform in CEP63 (A). In PREB (B) and PXN (C), activation causes increased inclusion of a cassette exon containing a stop site. Orange circles highlight regions increased in activated samples relative to resting and green circles highlight the opposite. (PDF) [file pone.0144409.s004.pdf]

**(A)**

**U2AF2  
RIPseq  
Schema**

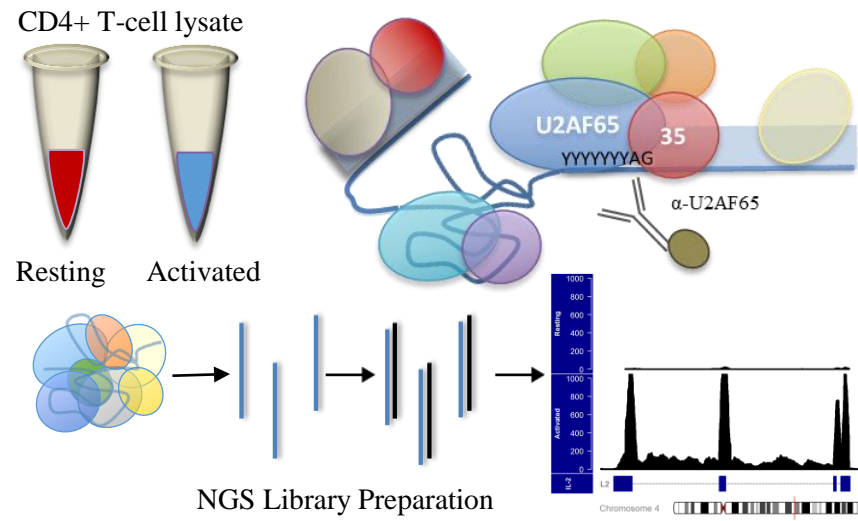

**(B)**

**CD4 T-cells**

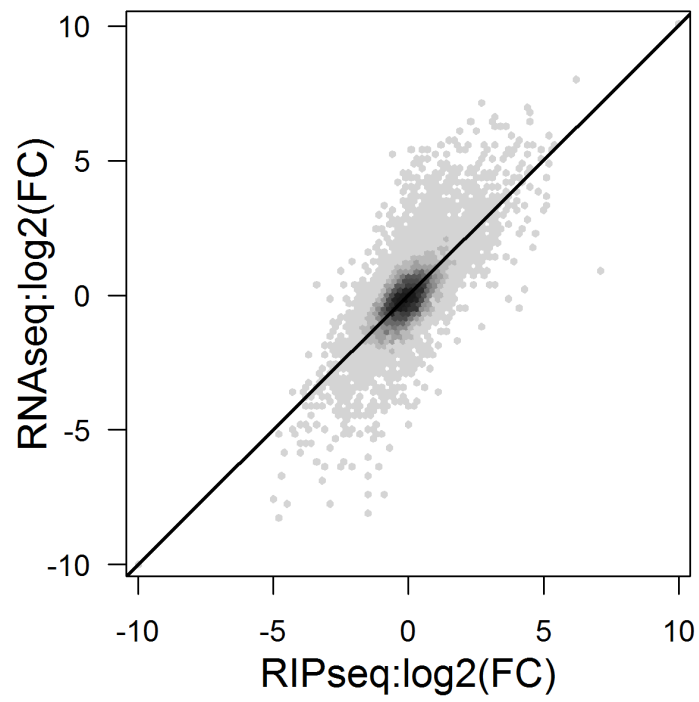

Supplement: S5 Fig — (A) Schematic of the U2AF2 RIP experiment followed by RNAseq. (B) Hexbin plot of U2AF2 RIPseq log2 fold changes (resting vs. activated) on the x-axis versus RNAseq log2 fold changes (resting vs. activated) on the y-axis shows that the methods have comparable results on a per gene basis. (PDF) [file pone.0144409.s005.pdf]

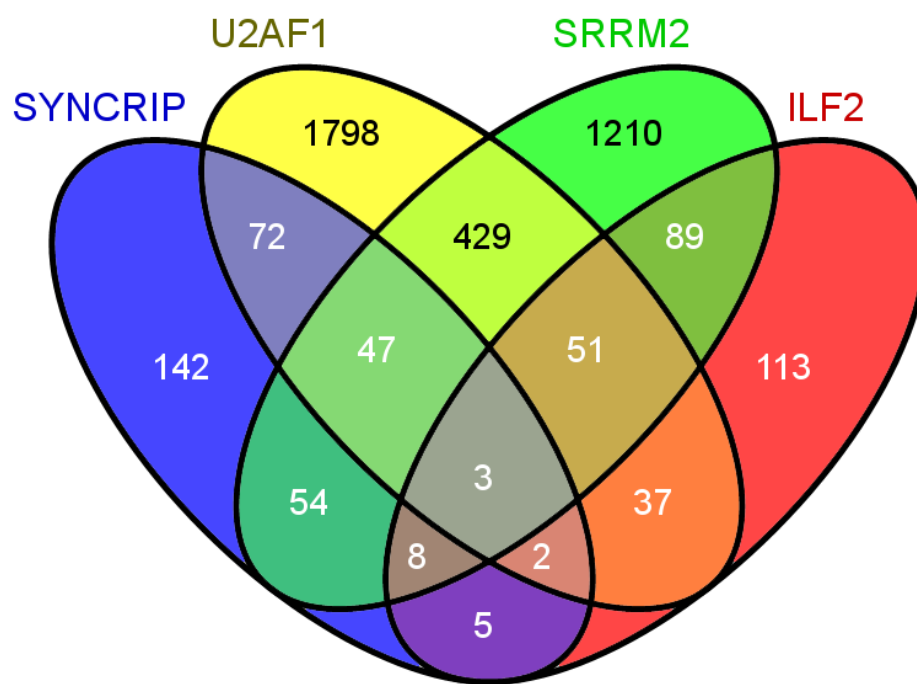

Supplement: S7 Fig — Overlap of differentially expressed genes in activated T cells with knockdown of U2AF1, SRRM2, SYNCRIP, and ILF2. (PDF) [file pone.0144409.s007.pdf]

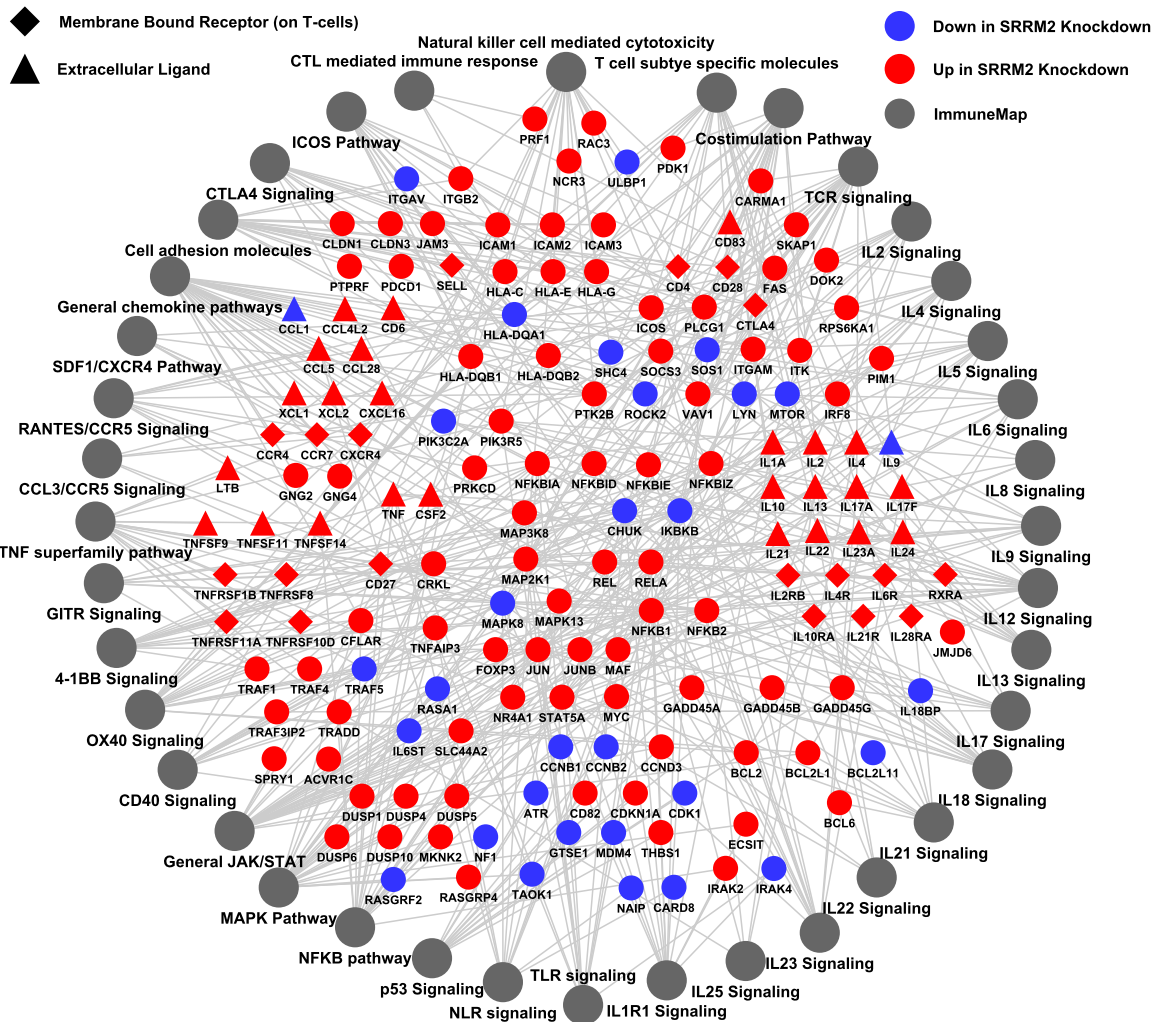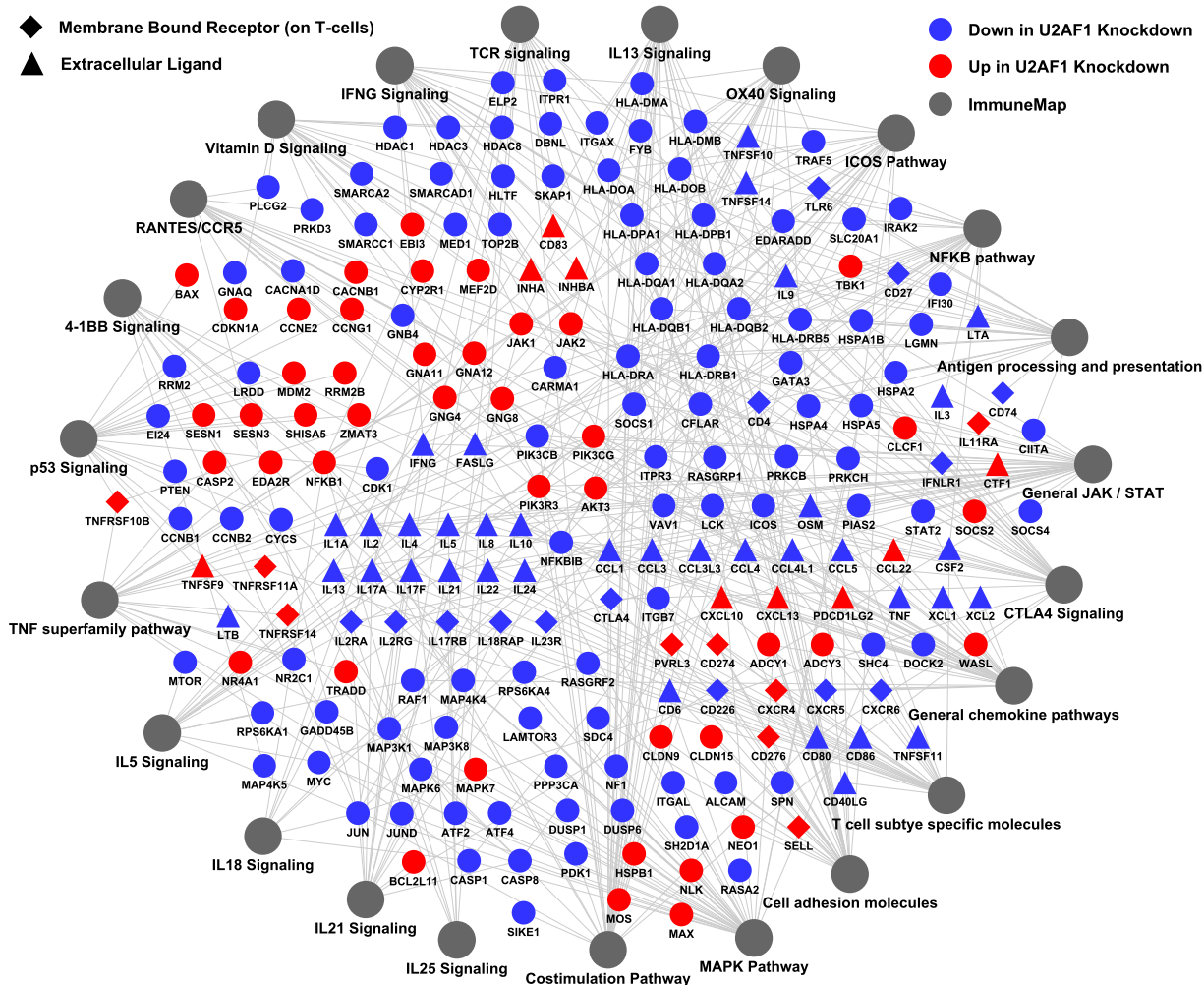

Supplement: S8 Fig — A Cytoscape network of enriched immune pathways (gray) containing differentially expressed genes (downregulated–blue, downregulated—red) in activated T cells with (A) SRRM2 and (B) U2AF1 knockdown. (PDF) [file pone.0144409.s008.pdf]

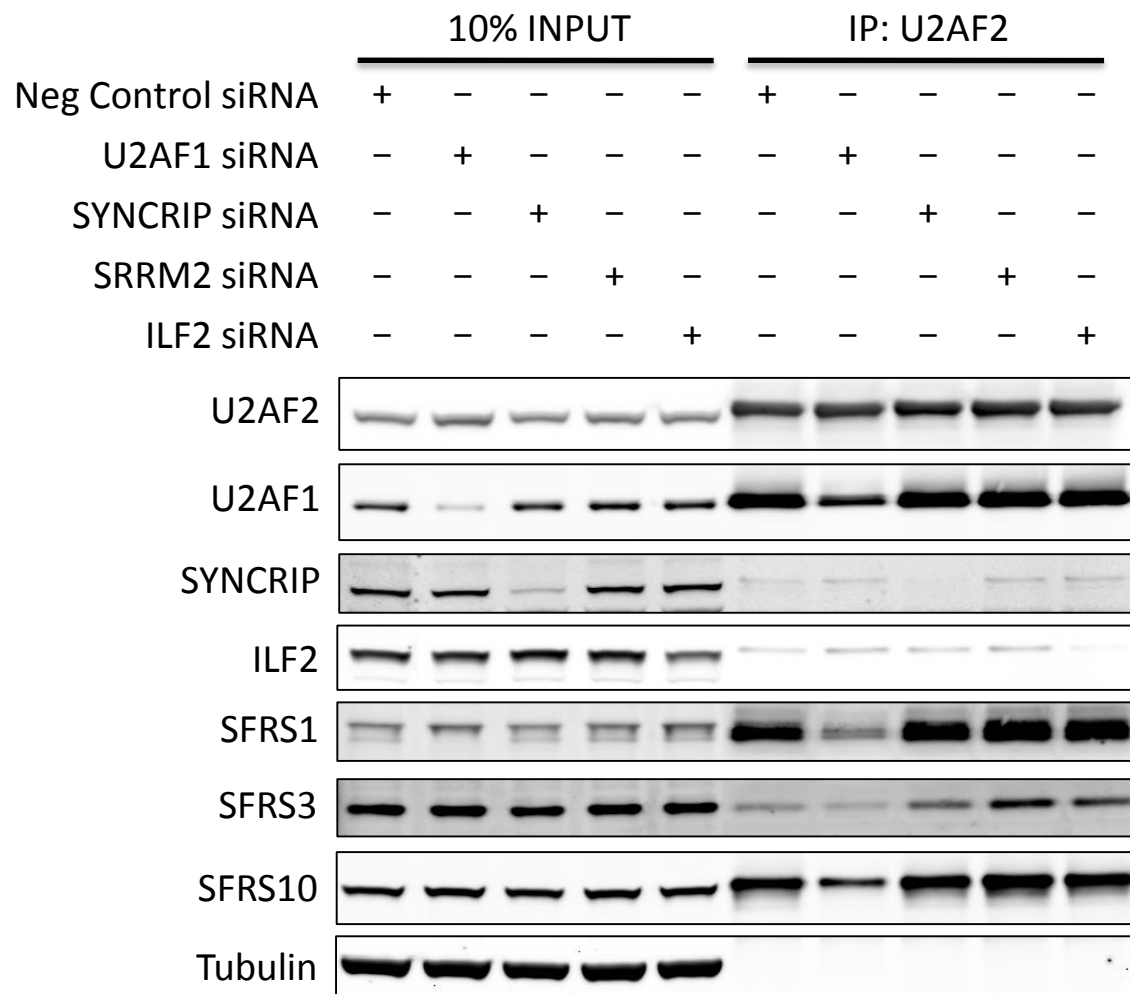

Supplement: S9 Fig — Immunoblot analysis for binding of select U2AF2 Interacting Proteins after immunoprecipitation with U2AF2 antibody in activated Jurkat T cell samples treated with the specified siRNA. (PDF) [file pone.0144409.s009.pdf]
