# Supplementary material for: The Activation-Induced Assembly of an RNA/Protein Interactome Centered on the Splicing Factor U2AF2 Regulates Gene Expression in Human CD4 T Cells
Source: PLoS One. 2015 Dec 7;10(12):e0144409. doi: 10.1371/journal.pone.0144409 (PMC4671683; doi:10.1371/journal.pone.0144409)
Supplement: S6 Fig — (A) Schematic of the U2AF2 RIP experiment followed by tripleTOF mass spectrometry. (B) Breakdown of the differential expression and splicing changes for the transcripts of U2AF2 interacting proteins. (C) Distribution of detected post-translational modifications in resting and activated cells after U2AF2 RIP-MS and the breakdown by type of modification in each group. (PDF) [file pone.0144409.s006.pdf]

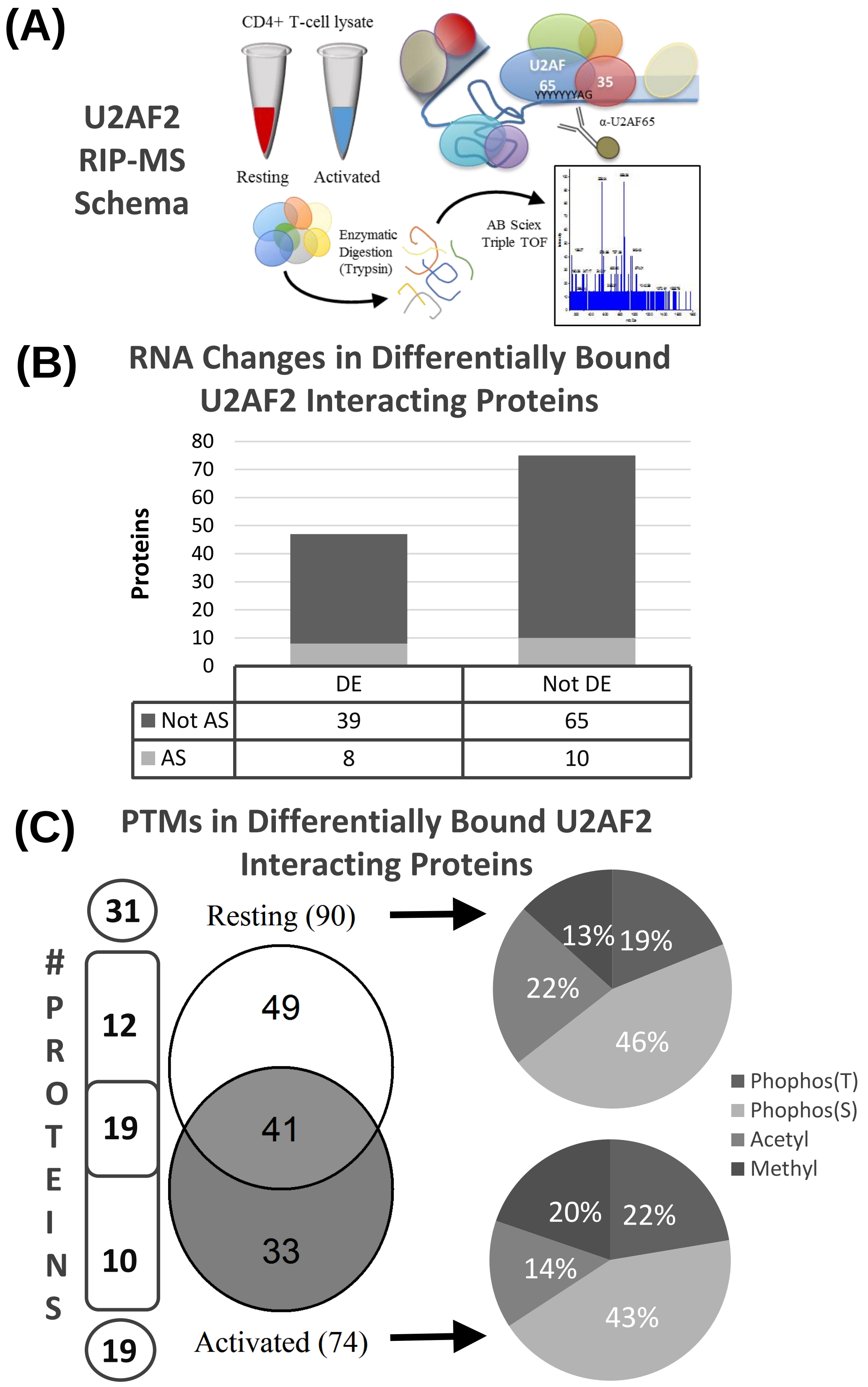
(A)
U2AF2
RIP-MS
Schema
CD4+ T-cell lysate
Resting
Activated
U2AF
65
35
YYYYYYYAG
α-U2AF65
Enzymatic
Digestion
(Trypsin)
AB Sciex
Triple TOF
(B)
RNA Changes in Differentially Bound U2AF2 Interacting Proteins
Proteins
80
70
60
50
40
30
20
10
0
DE
Not DE
Not AS
39
65
AS
8
10
(C)
PTMs in Differentially Bound U2AF2 Interacting Proteins
PROTEINS
31
12
19
10
19
Resting (90)
49
41
33
Activated (74)
13%
19%
22%
46%
Phophos(T)
Phophos(S)
Acetyl
Methyl
20%
22%
14%
43%
