# Supplementary material for: The Activation-Induced Assembly of an RNA/Protein Interactome Centered on the Splicing Factor U2AF2 Regulates Gene Expression in Human CD4 T Cells
Source: PLoS One. 2015 Dec 7;10(12):e0144409. doi: 10.1371/journal.pone.0144409 (PMC4671683; doi:10.1371/journal.pone.0144409)
Supplement: S2 Table — Statistics for enrichment of Gene Ontology–Biological Process categories (p-value < 0.05) of differentially expressed and alternatively spliced genes. (PDF) [file pone.0144409.s011.pdf]

S2 Table. Genes that are differentially expressed and alternatively spliced are enriched for GO categories.

Ontology Name(Ontology-ID)	Number Changed	Number Measured	Number in Ontology	Percent Changed	Percent Present	Z Score	FisherExactP
cell division (GO:0051301)	43	316	370	13.61	85.41	3.01	0.00457
immune system process (GO:0002376)	93	808	1562	11.51	51.71	2.75	0.00778
biological adhesion (GO:0022610)	34	250	732	13.6	34.15	2.66	0.0125
cell cycle(GO:0007049)	94	855	1052	10.99	81.27	2.28	0.02684
