# Supplementary material for: The Activation-Induced Assembly of an RNA/Protein Interactome Centered on the Splicing Factor U2AF2 Regulates Gene Expression in Human CD4 T Cells
Source: PLoS One. 2015 Dec 7;10(12):e0144409. doi: 10.1371/journal.pone.0144409 (PMC4671683; doi:10.1371/journal.pone.0144409)
Supplement: S3 Table — Statistics for enrichment of ImmuneMap pathways (adjusted p-value < 0.1) of differentially expressed and alternatively spliced genes. (PDF) [file pone.0144409.s012.pdf]

S3 Table. Genes that are differentially expressed and alternatively spliced are enriched for immune pathways.

Pathway	Total.Genes	Matched Genes	p-value	Adjusted p-value
Vitamin D Receptor Signaling	90	10	0.00276	0.0588
Costimulation Pathway	96	11	0.00135	0.0588
General chemokine pathways	190	16	0.00346	0.0588
p53 signaling	68	8	0.00503	0.0642
TCR Signaling	92	9	0.0101	0.0857
Eoxtaxin / CCL11 Signaling	106	10	0.00882	0.0857
