# Supplementary material for: The Activation-Induced Assembly of an RNA/Protein Interactome Centered on the Splicing Factor U2AF2 Regulates Gene Expression in Human CD4 T Cells
Source: PLoS One. 2015 Dec 7;10(12):e0144409. doi: 10.1371/journal.pone.0144409 (PMC4671683; doi:10.1371/journal.pone.0144409)
Supplement: S6 Table — Distribution of post-translational modifications in resting and activated T cells and the breakdown by type of modification for each protein. (PDF) [file pone.0144409.s015.pdf]

Table S6. Top 10 U2AF2 interactome members with detected post-translational modifications.

Protein	UNIPROT ID	# of PTMs	Resting Only	Activated Only	Resting/ Activated	Phospho (S)	Phospho (T)	Acetyl	Methyl (1,2,3)
**SRRM2**	Q9UQ35	39	12	13	14	29	10	0	0
**HNRNPA1**	P09651	9	2	3	4	3	0	1	5
**U2AF2**	**P26368**	**8**	**6**	**1**	**1**	**2**	**5**	**1**	**0**
**SFRS10**	P62995	5	2	0	3	1	2	0	2
**SFRS7**	Q16629	5	4	0	1	5	0	0	0
**ACIN1**	Q9UKV3	4	3	0	1	4	0	0	0
**SFRS1**	Q07955	4	2	0	2	3	0	0	1
**HMGB2**	P26583	3	3	0	0	0	0	3	0
**SFRS3**	P84103	3	3	0	0	0	0	2	1
**TRA2A**	Q13595	3	1	0	2	0	3	0	0
